# Identification and characterization of a rich population of CD34^+^ mesenchymal stem/stromal cells in human parotid, sublingual and submandibular glands

**DOI:** 10.1038/s41598-017-03681-1

**Published:** 2017-06-14

**Authors:** Padma Priya Togarrati, Robson T. Sasaki, Mohamed Abdel-Mohsen, Nuntana Dinglasan, Xutao Deng, Shivani Desai, Elaine Emmerson, Elizabeth Yee, William R. Ryan, Marcelo C. P. da Silva, Sarah M. Knox, Satish K. Pillai, Marcus O. Muench

**Affiliations:** 1 Blood Systems Research Institute, San Francisco, CA USA; 2 Department of Morphology and Genetics - Discipline of Descriptive and Topographic Anatomy, Federal University of São Paulo, Brazil, CEP USA; 3 Department of Medicine, University of California San Francisco, San Francisco, California USA; 4 The Wistar Institute, Philadelphia, PA USA; 5 Department of Cell and Tissue Biology, University of California, San Francisco, CA USA; 6 Division of Head and Neck Oncologic/Endocrine/Salivary Surgery, Department of Otolaryngology, University of California San Francisco, San Francisco, CA USA; 7 Department of Laboratory Medicine, University of California, San Francisco, CA USA

## Abstract

Mesenchymal stem/stromal cells (MSCs) play crucial roles in maintaining tissue homeostasis during physiological turnovers and injuries. Very little is known about the phenotype, distribution and molecular nature of MSCs in freshly isolated human salivary glands (SGs) as most reports have focused on the analysis of cultured MSCs. Our results demonstrate that the cell adhesion molecule CD34 was widely expressed by the MSCs of human major SGs, namely parotid (PAG), sublingual (SLG) and submandibular (SMG) glands. Further, gene expression analysis of CD34^+^ cells derived from fetal SMGs showed significant upregulation of genes involved in cellular adhesion, proliferation, branching, extracellular matrix remodeling and organ development. Moreover, CD34^+^ SMG cells exhibited elevated expression of genes encoding extracellular matrix, basement membrane proteins, and members of ERK, FGF and PDGF signaling pathways, which play key roles in glandular development, branching and homeostasis. *In vitro* CD34^+^ cell derived SG-MSCs revealed multilineage differentiation potential. Intraglandular transplantation of cultured MSCs in immunodeficient mice led to their engraftment in the injected and uninjected contralateral and ipsilateral glands. Engrafted cells could be localized to the stroma surrounding acini and ducts. In summary, our data show that CD34^+^ derived SG-MSCs could be a promising cell source for adoptive cell-based SG therapies, and bioengineering of artificial SGs.

## Introduction

Stem cells constitute an important facet of cell-based therapies in regenerative medicine. Mesenchymal stem/stromal cells (MSCs) are a group of specialized multipotent stem cells that have the ability to proliferate and differentiate into multiple mesodermal and non-mesodermal lineages and play key roles after injury and in the homeostatic maintenance of tissue architecture^1^. Widespread availability in various organs, ease of isolation and propagation under *in vitro* conditions, lack of ethical and teratoma formation concerns, extensive proliferative and differentiation abilities, paracrine secretory functions, and immunosuppressive behavior have rendered MSCs as the most sought after cell type in pre-clinical and clinical research fields^2, 3^. Additionally, MSCs play indispensable roles in the organogenesis and development of several epithelial organs including salivary glands (SGs)^4, 5^.

It has been shown that MSCs isolated from various tissue sources and donors possess differences in phenotype and multilineage differentiation abilities^6–8^. Further, the phenotypic expression profile also differs among *in situ* and *in vitro* cultured MSCs^9^. These limitations pose major hurdles in the widespread clinical utility of MSCs. Therefore, investigation of unique phenotypic markers in various organs could lead to generation of more homogenous pools of MSCs with defined downstream functional implications in a tissue-specific manner. Herein, we report on the extensive phenotypic and functional characterization of MSCs in all three major SGs in humans: parotid (PAGs), sublingual (SLGs) and submandibular (SMGs) glands.

SGs represent a group of exocrine organs with the primary function of synthesizing saliva. Saliva performs a plethora of crucial functions such as, mastication and digestion of food, protection of the oral cavity from bacterial infections, prevention of tooth decay, facilitating speech and induction of taste perception by stimulating taste buds^10, 11^. In humans, paired PAGs, SLGs and SMGs contribute to >90% of the total saliva secretion.

SGs originate from the embryonic ectodermal epithelial progenitors around the 6^th^ to 8^th^ week of gestation. By the 28^th^ week, a series of key physiological events lead to the development of mature SGs that can secrete saliva at birth^12, 13^. Complex reciprocal interactions between epithelial, mesenchymal, vascular and neuronal progenitors lead to the organogenesis and development of SGs. Embryonic mesenchyme, through secretion of growth and signaling factors, provides essential molecular cues to the developing epithelial progenitors at various stages of glandular development and histodifferentation^5^. Functional significance of MSCs has been further demonstrated through recombination experiments between epithelial and mesenchymal progenitors, which have established that signals from mesenchyme regulate the branching pattern and type of saliva, either serous or mucus, secreted by the developing acinar cells in SGs^14–16^.

Various groups have studied expression of stem/progenitor cell markers such as, aldehyde dehydrogenase (ALDH), c-kit (CD117), CD24, CD29, CD34, CD44, CD49f, CD90, CD133 and CD166 in SG cell aggregates known as ‘salispheres’ and in cell monolayers grown under culture conditions, and have further demonstrated regenerative and reparative functions of these cells in various mouse models of SG damage^17–23^. However, these cultured cells were derived from whole cell isolates of SGs containing mixtures of cells of multiple lineages. The phenotypic markers identifying MSCs in freshly isolated SGs remain largely uncharacterized. Moreover, molecular nature of primitive embryonic mesenchyme from SMGs has been studied in mice, but little is known about the gene expression profile of the mesenchyme of human fetal SMGs^14–16^.

The current study investigated human PAGs, SLGs and SMGs to understand (i) the phenotypic expression profile of MSCs in freshly collected SGs as compared to age-matched bone marrow (BM), (ii) *in situ* localization and functional properties of a population of SG-MSCs enriched for the adhesion molecule CD34, (iii) the gene expression profile of sorted CD34^+^ and CD34^−^ cells derived from midgestation SMGs, (iv) *in vitro* growth and multilineage differentiation potential of cultured MSCs isolated from sorted CD34^+^ SG cells, and (v) *in vivo* functional ability of CD34^+^ cell-derived MSCs, when transplanted intraglandularly into immunodeficient NOD.Cg-*Prkdc* ^*scid*^ *Il2rg* ^*tm1Wjl*^/SzJ (NSG) mice. Insights from this study will help in facilitating development of effective cellular therapies for xerostomia, various SG diseases and malignancies.

## Results

### Human fetal PAGs, SLGs and SMGs contain a rich population of CD34^+^ MSCs

**Figure 1:**
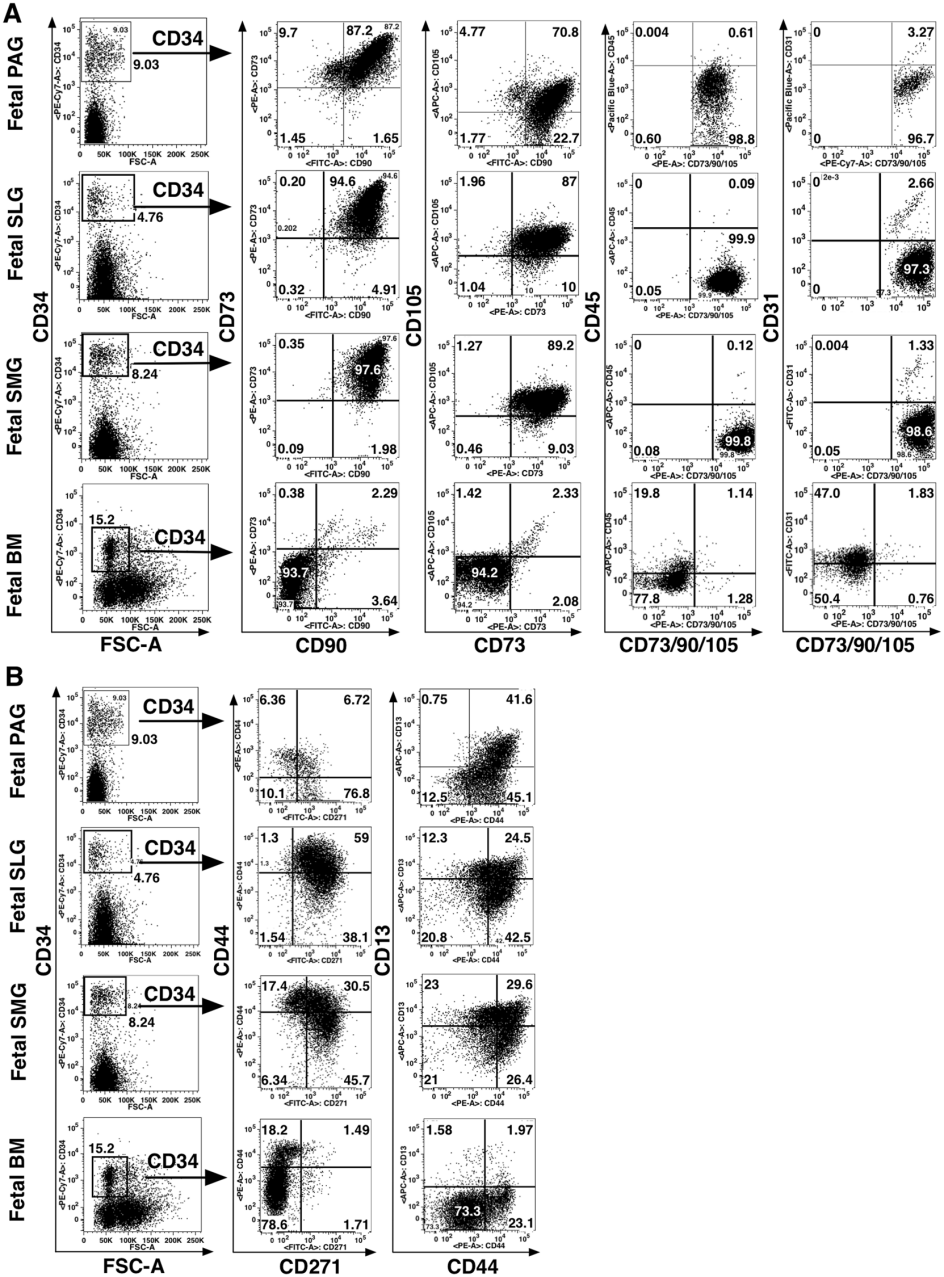
Characterization of mesenchymal progenitors in fetal PAGs, SLGs, SMGs and BM-MNCs. (**A**) Expression of CD73, CD90, CD105, CD45 and CD31 on gated total CD34^+^ cells. (**B**) Analyses of expression of CD44, CD271 and CD13 on total CD34^+^ cells.

Analysis of expression of various lineage specific stem/progenitor cell surface markers in fetal SGs (16–24 weeks’ gestation), including MSC markers defined by the International Society for Cellular Therapy (ISCT) (listed in Table S1), showed that expression of MSC specific markers, CD73, CD90 and CD105 was higher in fetal PAGs, SLGs and SMGs in comparison to bone marrow mononuclear cells (BM-MNCs) (Figure S1A). Interestingly, the cell surface marker CD34, which is usually highly expressed by hematopoietic precursors, was also found to be highly expressed on fetal SGs (Figs 1A and S1B). The majority of CD34^+^ SG cells were positive for CD73, CD90 and CD105; they also expressed other MSC associated markers CD271, CD44 and CD13 (Figs 1B and S1B). Significantly high expression of CD73, CD90, CD105 and CD13 was observed on CD34^+^ SG cells as compared to BM CD34^+^ cells (P < 0.05, Mann-Whitney test) (Figs 1 and S1B). Similar to typical MSCs, CD34^+^ SG cells expressed little CD45, CD31, CD19, and HLA-DR (Figs 1A and S2). These results indicate the rich mesenchymal nature of the fetal SG tissues as compared to the BM, which is predominantly comprised of hematopoietic cells.

**Figure 2:**
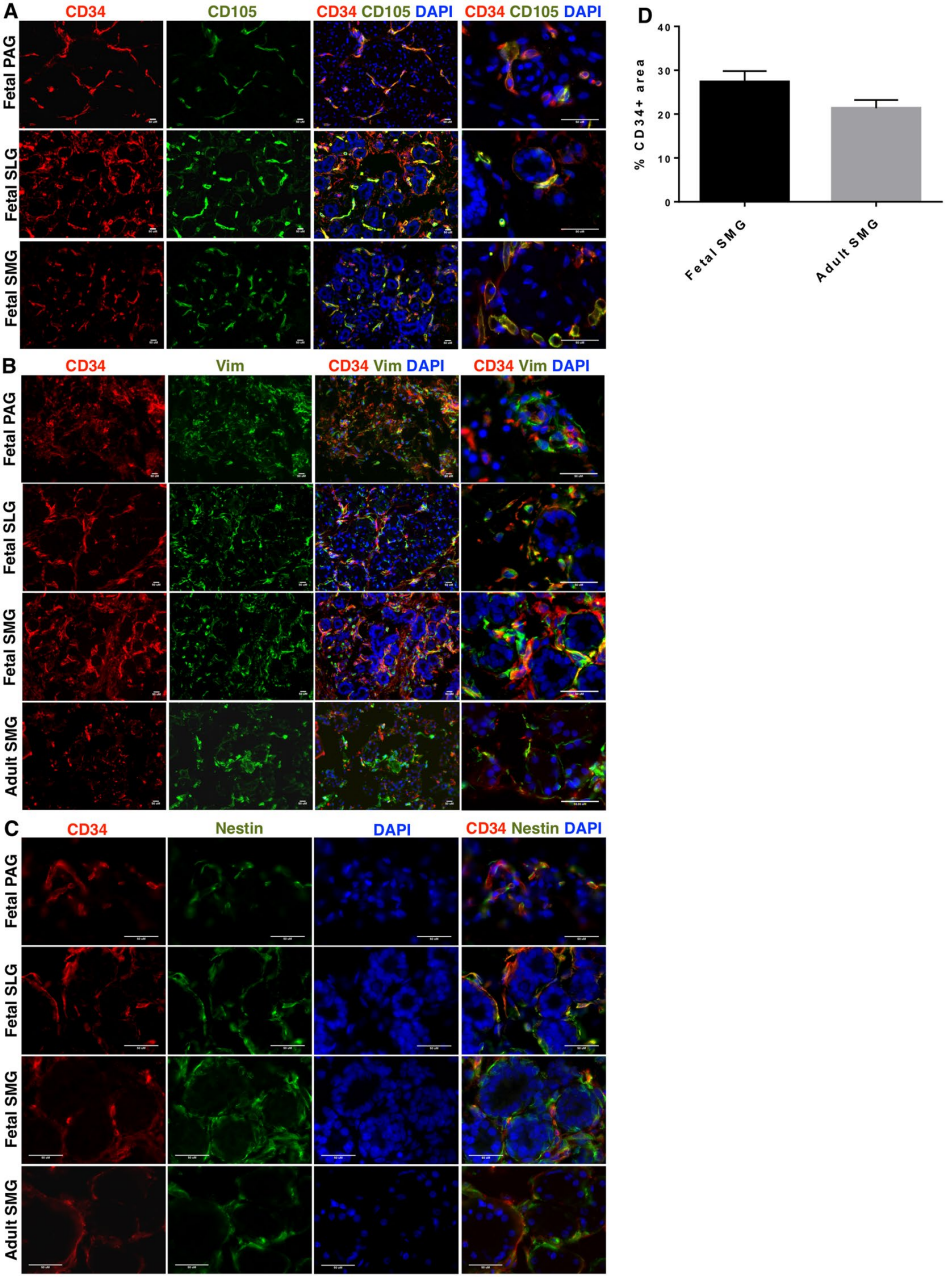
*In situ* localization of (**A**) CD34 (red) and CD105 (green) in fetal SGs (**B**) CD34 (red) and vimentin (vim) (green) (**C**) CD34 (red) and nestin (green) in fetal SGs and adult SMGs. All scale bars represent 50 µM. (**D**) Quantitative differences in the expression of CD34 between fetal and adult SMGs. Error bars represent mean ± SEM.

Further, *in situ* localization and distribution of CD34^+^ cells was investigated in the context of other known lineage specific stem/progenitor and committed SG markers CD133 (prominin-1), K5 (cytokeratin 5) and c-Kit (CD117), as well as lineage markers for endothelial (CD31/PECAM-1), myoepithelial [α-smooth muscle actin (α-SMA) and calponin], epithelial [EpCAM (epithelial cell adhesion molecule) and K18 (cytokeratin 18)] and neuronal (CD56/NCAM) cells. Immunohistochemistry results showed that the majority of the CD34^+^ cells were widely distributed in the stroma lining the acini and ducts of fetal SGs, and co-expressed MSC markers CD105 (Fig. 2A), vimentin (Fig. 2B) and nestin (Fig. 2C). Likewise, CD34 expression was confined to the stromal regions of the adult SMGs. Quantitative comparison of the differences in the expression of CD34 on fetal and adult SMGs showed higher expression of CD34 in fetal SMGs as compared to adult SMGs (Fig. 2D).

**Figure 3:**
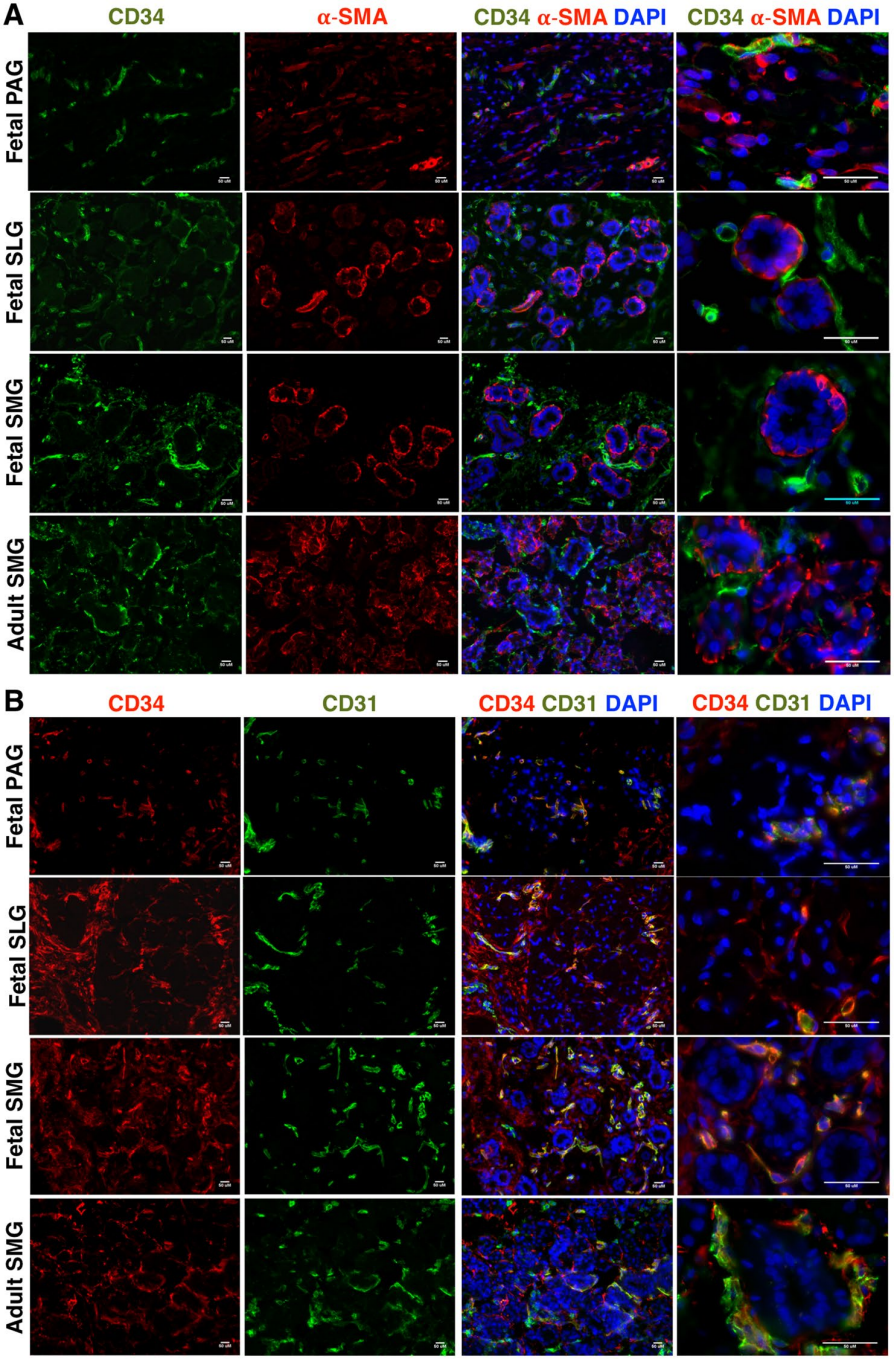
*In situ* localization of (**A**) CD34 (green) and α-SMA (red) (**B**) CD34 (red) and CD31 (green) in fetal SGs and adult SMGs. All scale bars represent 50 µM.

**Figure 4:**
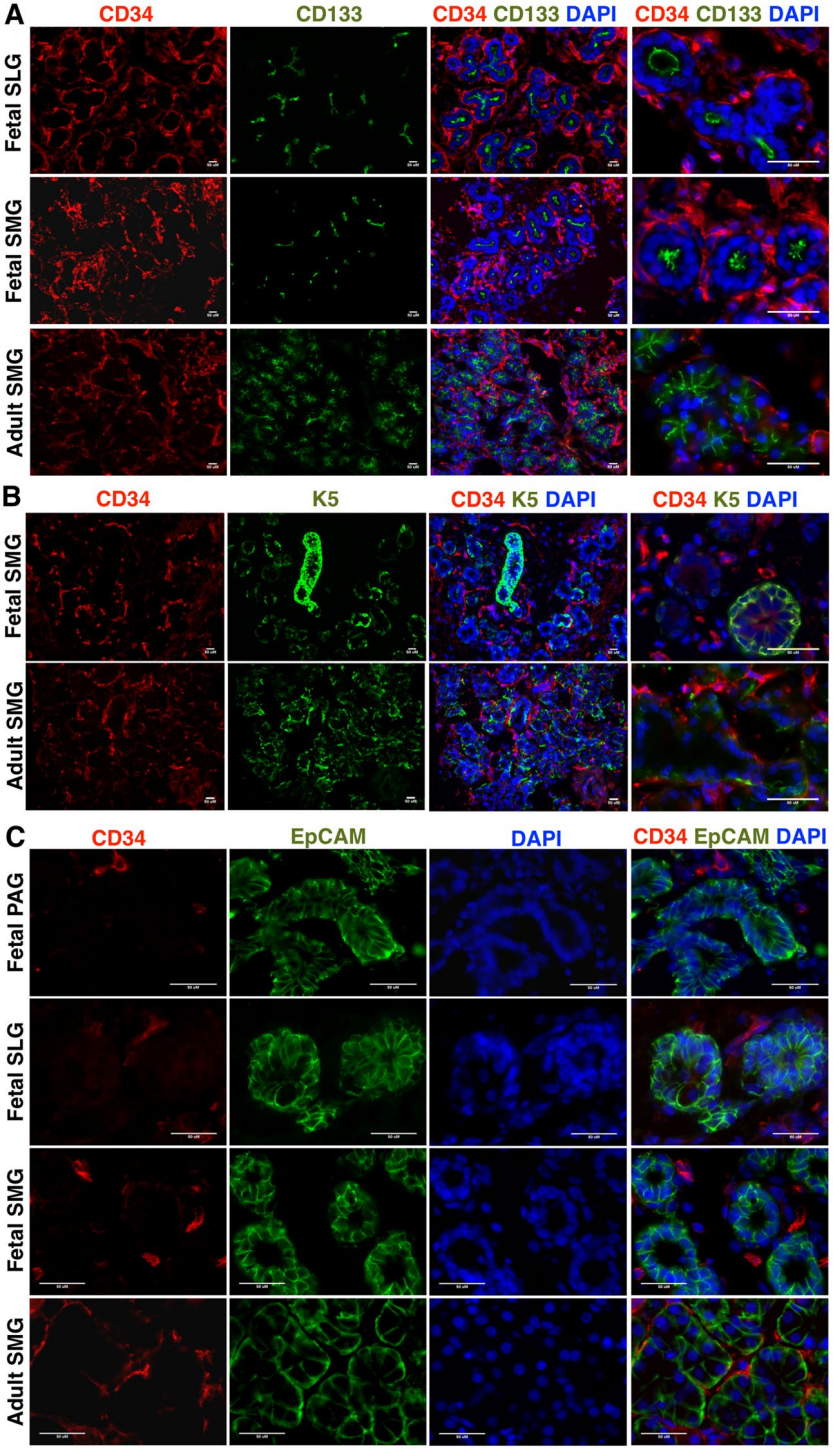
*In situ* localization of (**A**) CD34 (red) and CD133 (green) (**B**) CD34 (red) and K5 (green) (**C**) CD34 (red) and EpCAM (green) in fetal SLGs, SMGs, and adult SMGs. All scale bars represent 50 µM.

A rare sub-population of CD34^+^ cells co-expressing CD105 was found in intralobular ducts (Figure S3A). However, further investigation of co-expression of CD34 and epithelial cell marker cytokeratin 18 (K18) (Figure S3B,C) revealed that CD34^+^ cells did not express the aforementioned epithelial marker. This finding further supported the MSC specific expression of CD34. *In situ* findings further reinforced the fact that CD34^+^ cells in SGs were distinct from myoepithelial cells expressing α-SMA (Fig. 3A) and calponin (Figure S3D). *In situ* localization results showed that CD31 expression co-localized with some CD34^+^ cells in fetal SGs. However, the majority of CD34 expression was associated with the CD31^−^ stromal tissues of fetal SGs (Fig. 3B). Further CD34^+^ cells did not express the epithelial progenitor makers CD133 (Figs 4A and S4A,B), K5 (Fig. 4B), EpCAM (Fig. 4C), c-Kit cells (Figure S4A,B) and neuronal marker CD56 (Figure S5). CD133 expression marked the presence of luminal epithelial progenitors of SLGs and SMGs (Figs 4A and S4B). K5 was widely distributed in the ductal and myoepithelial progenitors of the fetal SMGs (Fig. 4B). Expression of CD133 and K5 was found to be prominent in more branched SMGs and SLGs (Fig. 4A,B). CD56 was detected on the neural cells of the acini and ducts (Figure S5C), and in the nerve bundles of fetal SGs (Figure S5D).

In accordance to the findings in fetal SGs, vimentin (Fig. 2B), α-SMA (Fig. 3A), calponin (Figure S3D), CD31 (Fig. 3B), CD133 (Fig. 4A), K5 (Fig. 4B) and CD56 (Figure S5) expression could also be detected in adult SMGs. As expected, cells expressing the aforementioned lineage markers were more developed and differentiated in adult SMGs as compared to fetal SGs. Overall, these findings suggest that CD34^+^ cells represent a putative MSC population in SGs.

### CD34^+^ SMG cells express genes crucial for early gland development, branching and homeostasis

Studies have shown that primitive SG mesenchyme plays a crucial role in guiding epithelial progenitor budding, morphogenesis and differentiation by providing essential molecular cues in the form of extracellular matrix (ECM)/basement membrane proteins and secretory growth factors^4, 5, 24^. Mesenchyme also contributes to controlling the pattern of epithelial branching, and hence, in deciding the specification of the gland type. The importance of tissue-specific mesenchyme in branching and cellular specification was further demonstrated in recombination experiments showing transformation of pituitary epithelium into SMG phenotype when the former was recombined with SMG mesenchyme^16^. In various murine studies, embryonic SMG tissue has served as a model system to understand various stages of primitive glandular development, branching morphogenesis, canalization and cytodifferentiation^4, 5, 24^. In humans, SMGs contribute to 70–75% of the total saliva secretion in adults^25^. Thus, owing to the physiological importance of SMGs, we carried out comparative gene expression analysis in sorted CD34^+^ and CD34^−^ cells isolated from fetal SMGs (n = 3, 22 weeks’ gestation).

**Figure 5:**
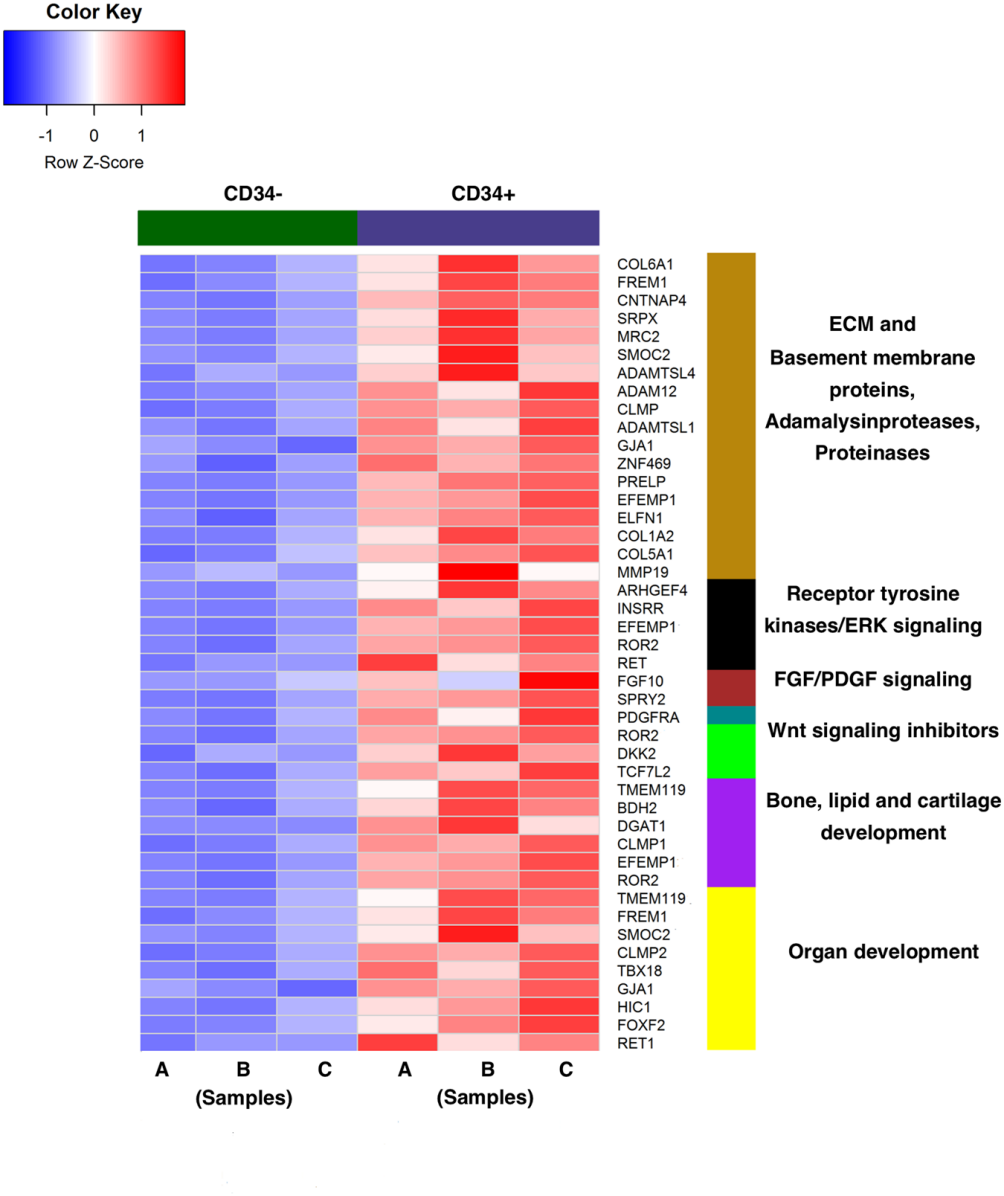
RNA sequence profiling of CD34 cells derived from human fetal SMGs. Heat map shows genes significantly upregulated in CD34^+^ population as compared to CD34^−^ cells.

Differentially-expressed genes in CD34^+^ versus CD34^−^ populations were selected based on the expression fold ratio value being ≥2, paired t-test P-value < 0.01, and normalized gene expression values being 3 in at least one of the samples. Of the total 262 differentially expressed genes, 46 genes were found to be upregulated and 216 genes were downregulated in CD34^+^ cells as compared to CD34^−^ cells. The majority of the genes that were significantly enriched in CD34^+^ cells possessed functions associated with MSCs such as genes encoding proteins specific for ECM, basement membrane, ECM assembly and remodeling, regulating cell adhesion, cell-ECM interactions, cell proliferation, proteases, matrix metalloproteinases, and components of signaling pathways relevant to early gland branching and development (Fig. 5). High expression of some of these genes in CD34^+^ cells was additionally validated by quantitative real-time PCR analysis (Figure S6A).

**Figure 6:**
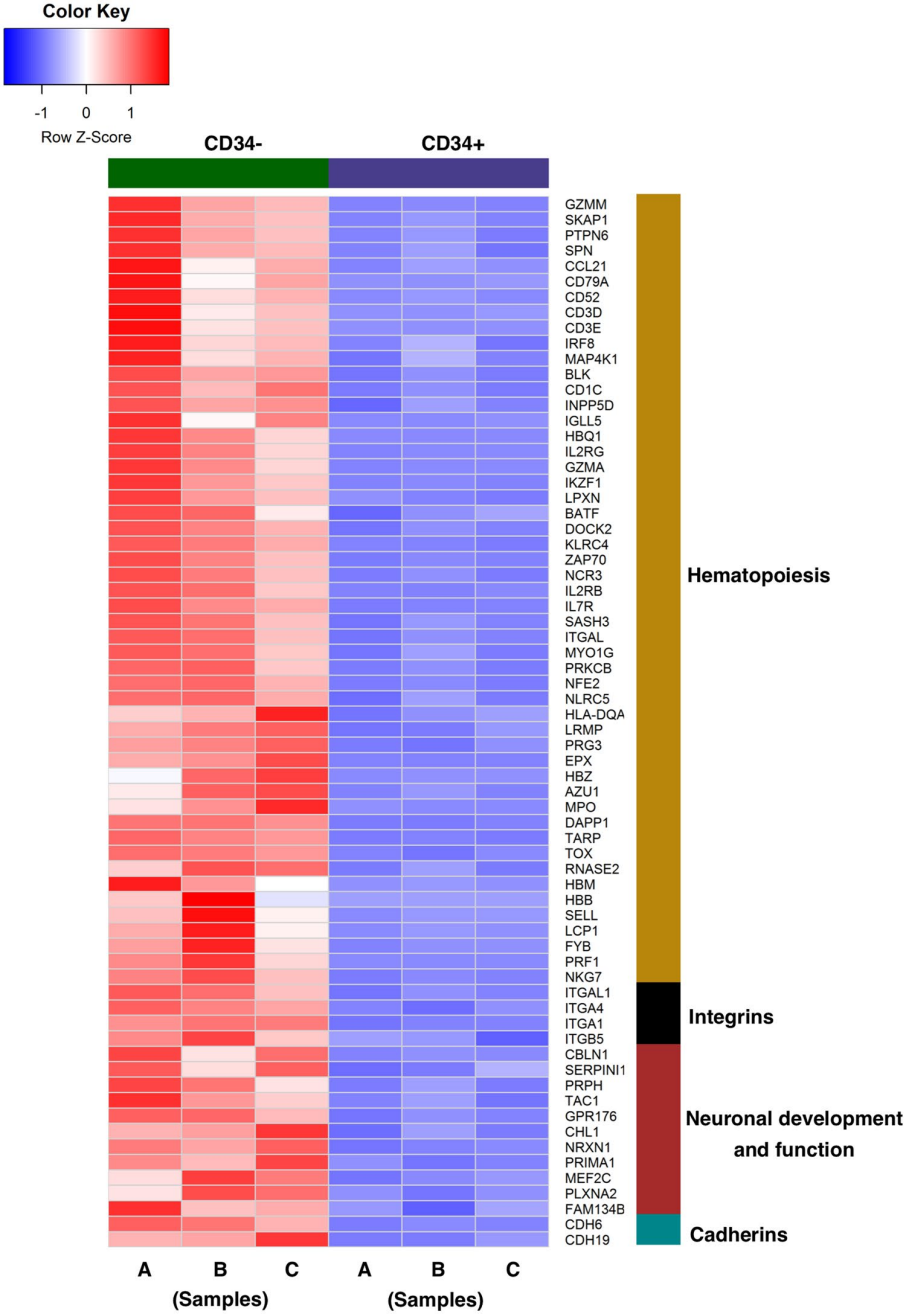
RNA sequence profiling of CD34 cells derived from human fetal SMGs. Heat map shows genes that are significantly downregulated in CD34^+^ population as compared to CD34^−^ cells.

On the other hand, functional annotation of genes downregulated in CD34^+^ cells revealed enrichment of genes primarily involved in hematopoiesis and neuronal development (Fig. 6). Downregulated genes in CD34^+^ cells also included members of the integrin (*ITGAL1*, *ITGA4*, *ITGA1* and *ITGB5*) and cadherin (*CDH6* and *CDH19*) families of proteins that are usually expressed on epithelial cells. Interestingly, the gene encoding the co-receptor of semaphorin proteins, plexin A2 (*PLXNA2*), that has been shown to be expressed on epithelial cells^26^, was found to be downregulated in CD34^+^ cells (Fig. 6). Moreover, genes that are expressed on epithelial cells such as *GZMA*, *PRIMA1*, *NRXN1*, *CHL1*, *AZU1*, *LCP1*, *PRKCB* and *LRMP* were found to be upregulated on the CD34^−^ subset.

Sequeira *et al*., have reported functional association of ECM proteins such as collagens and fibronectins in branching morphogenesis and induction of differentiation in the embryonic SMG development of mice^27^. In our study, it was revealed that CD34^+^ cells significantly upregulated expression of genes encoding extracellular matrix (ECM) and basement proteins such as *COL6A1*, *COL1A2*, *COL5A1*, *FREM1*, *PRELP* and *EFEMP1* (Fig. 5). *ELFN1* and *MRC2* genes that contain domains specific for ECM proteins such as fibronectin and fibulin domains, and *ZNF469*, that encodes proteins homologous to collagen were found to be enriched in the CD34^+^ population (Fig. 5). ECM degradation and remodeling, which are important prerequisites for cell migration and branching, are regulated by the action of proteases^28^. Proteases from both ADAM and matrix metalloproteinase protein families such as *ADAMTSL1*, *ADAMTSL4*, *ADAM12* and *MMP19* were found to be upregulated in CD34^+^ cells (Fig. 5).

Employing knock-out mouse models and function-blocking antibodies, various signaling pathway components such as FGF10, receptor tyrosine kinases such as EGFR and PDGFRA have been identified as key regulators of epithelial cell clefting and proliferation during branching morphogenesis, and induction of cytodifferentiation during the development of SMGs^29–31^. Interestingly, *FGF10*, *PDGFRA* and genes encoding several receptor tyrosine kinase family proteins were found to be considerably upregulated in CD34^+^ cells (shown in Fig. 5) (some of these comparisons were significant using a standard P < 0.05 threshold, but were not significant using our stringent P < 0.01 cutoff). A recent study showed that canonical Wnt signaling plays an important role in the proliferation and expansion of epithelial progenitors^32^. Interestingly, we observed enrichment of inhibitors of canonical Wnt signaling such as, *ROR2* and *DKK2* in CD34^+^ cells. Higher expression levels of another Wnt pathway member *TCFL2*, which can act either as an activator or repressor depending on the presence of *CTNNB1* was seen in CD34^+^ cells. Elevated expression of genes involved in the development and differentiation of bone, lipid and cartilage was also found in the enriched CD34^+^ cell transcriptome (Fig. 5). Additionally, CD34^+^ SMG cells showed upregulation of genes such as, *FREM1*, *SMOC2*, *HIC1*, *FOXF2* etc. involved in the development of tissues/organs such as neural crest, face, lung and intestine (Fig. 5).

We also compared the gene expression profile of CD34^+^ cell derived SG-MSCs to that of BM-MSCs isolated based on CD271 (NGFR) that has been shown to be expressed by the MSCs of BM^33^. Thus, expression of some of the genes such as, *COL6A1*, *COL5A1*, *FGF10*, *PDGFRA*, *ROR2*, *GJA1*, *SMOC2* and *HIC1* that were highly expressed in CD34^+^ SMG cells were analyzed in positively enriched CD271^+^ BM-MSCs. Interestingly, expression levels of the genes encoding ECM and gap junction proteins, e.g. *COL6A1*, *COL5A1* and *GJA1*, were found to be high in both BM- and SG-MSCs (Figure S6B). Similarly, *SMOC2* and *ROR2*, genes that have been reported to be expressed by MSCs^34, 35^, were also found to be enriched in CD271^+^ BM and CD34^+^ SG MSCs^34, 35^. However, the growth factor genes *FGF10* and *PDGFRA* that play an important role in glandular development and branching, and *HIC1* that plays an important role in craniofacial development, were upregulated in CD34^+^ SG-MSCs as compared to CD271^+^ BM-MSCs (Figure S6B). These findings highlighted the similarities and differences in the MSCs present in different tissues, and significance of intrinsic tissue-specific mesenchymal properties of CD34^+^ SG-MSCs.

### CD34^+^ cells from fetal PAGs, SLGs and SMGs can expand and form MSC monolayers with multilineage differentiation potential under *in vitro* culture conditions

**Figure 7:**
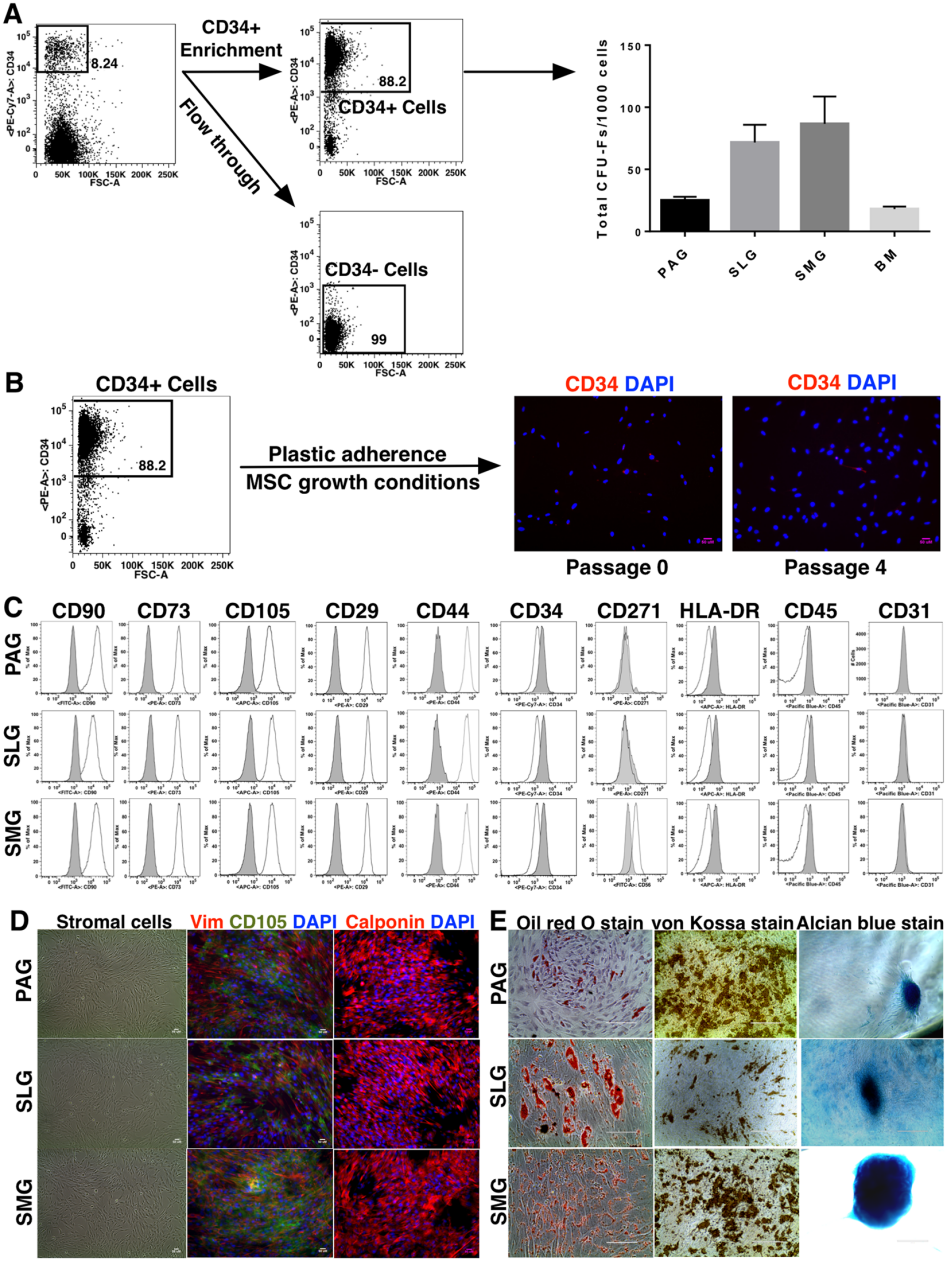
Phenotypic and functional characterization of CD34^+^ cell derived MSCs of fetal SGs (**A**) positive selection of CD34^+^ cells, and CFU-Fs formed per 1000 CD34^+^ cells (**B**) CD34 expression on cultured MSCs [Passages (P) 0 and 4]. All scale bars represent 50 µM. (**C**) Phenotypic analyses of MSC specific cell surface markers on cultured MSCs (P3 to 12), isotype control (grey). (**D**) Bright field stromal cell images, and expressions of vimentin (green), CD105 (red) and calponin (red) on cultured MSCs. Nuclei was stained in DAPI (blue). All scale bars represent 50 µM. (**E**) Multilineage differentiation into adipogenic (oil Red O staining), osteogenic (von Kossa staining) and chondrogenic (Alcian blue staining) lineages (P3 to 12). All scale bars represent 100 µM.

Mesenchymal properties of the CD34^+^ cells were further investigated by culturing them *in vitro* under MSC-specific growth conditions. Colony forming unit-fibroblast (CFU-F) assay results confirmed that the purified CD34^+^ cell fraction possessed the potential to form fibroblast colonies, whereas CD34^−^ cells formed very few colonies (Fig. 7A). Additionally, the number of CFU-Fs formed was higher in fetal SGs as compared to age-matched BM (Fig. 7A). Expression of CD34 gradually decreased as the sorted CD34^+^ cells were cultured on plastic dishes, and was lost completely by passages 3 or 4 (Fig. 7B). Similarly, CD271 expression was lost on the cultured MSCs (Fig. 7C). CD34^+^ cells from PAGs, SLGs and SMGs formed monolayers with long spindle-shaped cells and displayed typical stromal cell phenotype; these cells showed positive expression of vimentin, calponin, CD73, CD90, CD105, CD29 and CD44 markers, and were negative for HLA-DR, CD45, CD31 (Fig. 7C,D), CD33, CD19 and CD14 (Figure S7A). Additionally, CD34^+^ cell derived fetal SG-MSCs showed multilineage differentiation ability and could differentiate into adipocytes, osteocytes and chondrocytes (Figs 7E and S7B). These results indicated that CD34^+^ cells isolated from fetal SGs could form MSCs with extensive growth and multilineage differentiation abilities under *in vitro* culture conditions.

### Human fetal CD34^+^ cell derived SG-MSCs can engraft and differentiate when transplanted intraglandularly into NSG mice

**Figure 8:**
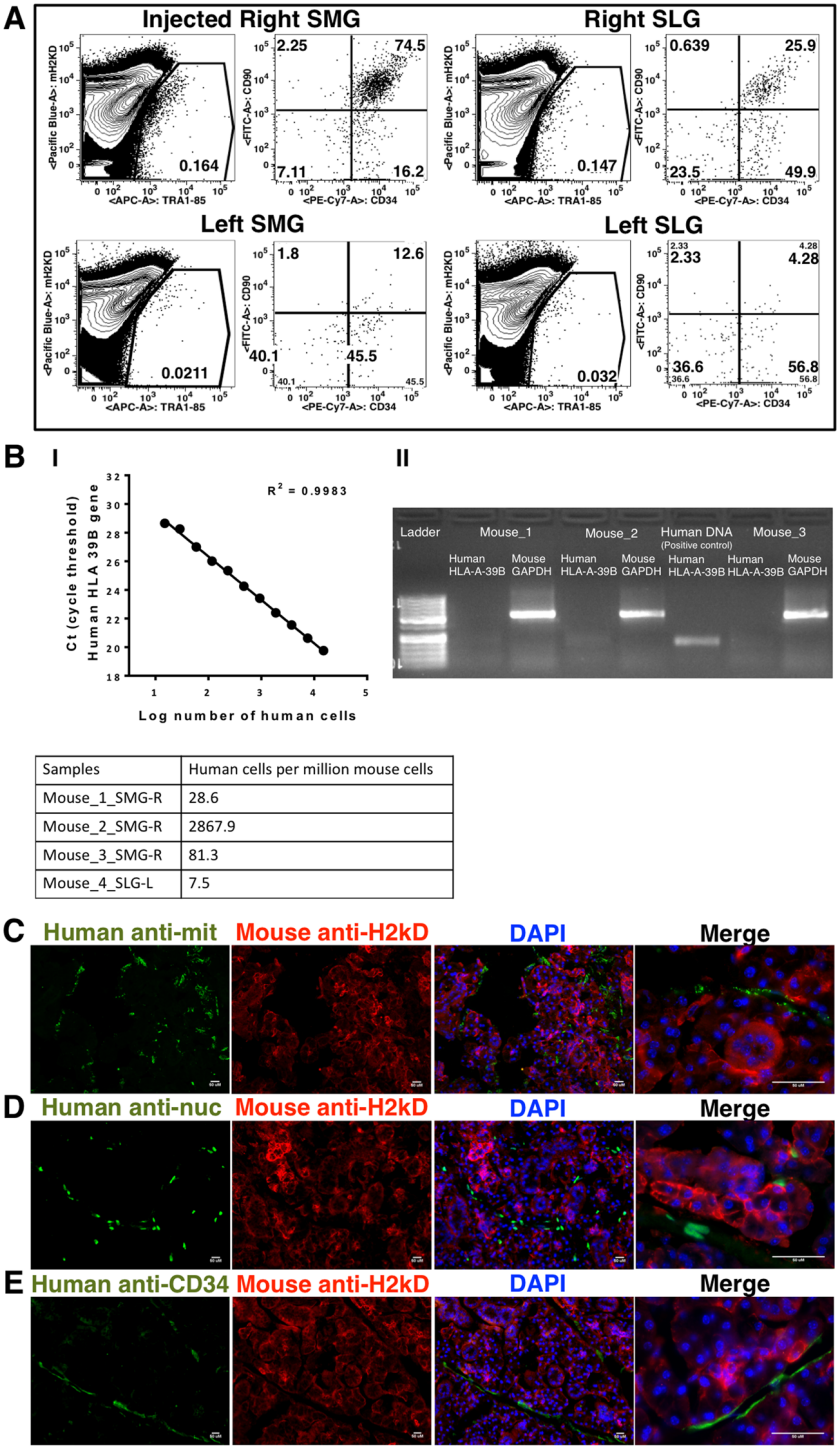
*In vivo* engraftment and functional characterization of PAG, SLG and SMG derived MSCs in NSG mice (**A**) engraftment and migration of injected human cells in paired mice SMGs and SLGs. Human cells were stained with pan human TRA1-85 (CD147) antibody. Mouse cells were detected by mouse specific H-2K^d^ staining. (**B**) Detection and quantification of human cell engraftment by quantitative real-time PCR. (**C**) Immunohistochemical detection of engrafted human cells using pan human marker, human anti-mitochondrial antibody (green) and mouse H-2K^d^ (red) in SMG of mice. (**D**) Immunohistochemical detection of engrafted human cells using pan human marker, human anti-nuclei antibody (green) and mouse H-2K^d^ (red) in SLG of mice. (**E**) Analysis of expression of human CD34 (green) on engrafted human cells in mice SMG using mouse H-2K^d^ (red). Nuclei was stained with DAPI (blue). All scale bars represent 50 µM.

Based on the findings from the phenotypic and molecular gene expression analyses, and *in situ* and *in vitro* observations that CD34^+^ cells in fetal SGs manifest phenotypic and functional attributes pertinent to MSCs, we further investigated their ability to engraft and repopulate *in vivo*, when transplanted intraglandularly into immunodeficient mice. Culture grown CD34^+^ cell-derived MSCs (passages 3 to 8) from human fetal PAGs, SLGs and SMGs were injected at three cell dosages (0.5, 1 and 2 million cells), and mice were analyzed for human cell engraftment 7, 15 and 30 days post-transplantation. Even though BM CD34^+^ cells possessed less fibroblast colony forming ability than SGs, through prolonged culture, we could also expand sufficient numbers of cells to be transplanted in mice to compare *in vivo* functional efficiency of fetal SG-MSCs over BM-MSCs. In addition to the injection site, transplanted MSCs could also be detected in the uninjected contralateral and ipsilateral SLGs and SMGs (Figs 8A and S8). Engrafted human cells were evaluated by flow cytometry using the pan-human marker TRA1-85 (CD147) to discriminate them from mouse cells. The presence of engrafted human cells in mice SLGs and SMGs was further confirmed by a highly sensitive human specific DNA based quantitative real-time PCR assay using human cell specific human leukocyte antigen (HLA)-A- 39B primer^36^. This assay demonstrated that injected human cells could persist in mouse tissues even 7, 15 and 30 days post-transplantation (Fig. 8B). Moreover, engrafted human cells could be localized to the mouse SLGs and SMGs using human-specific anti-mitochondrial and nuclear antibodies (Fig. 8C,D). None of the injected human cells were detected in the liver and lungs (Figure S9A,B). Immunohistolocalization results in the SLGs and SMGs of transplanted mice showed that engrafted human CD34^+^ cells were present in the stromal tissues surrounding acini and excretory ducts (Fig. 8E). Lineage analysis of engrafted cells by combinatorial approaches of flow cytometry and immunohistolocalization showed that SG-MSCs that had lost CD34 expression when grown in culture, re-expressed CD34 *in vivo* (Figs 8A,E and S9C). The engrafted human cells also expressed CD90 but were negative for CD31 expression (Figure S9C). No clear correlation was found between levels of engraftment, cell dose and type of SG-MSCs injected. Intriguingly, no clear engraftment of BM CD34^+^ derived MSCs was seen in the transplanted mice SLGs and SMGs (Figure S9D). These results indicated that, even under normal physiological conditions, without a major damage-induced trigger, tissue specific CD34^+^ cell-derived SG-MSCs have the ability to engraft, survive and repopulate the SGs in a tissue-specific fashion.

## Discussion

SGs are comprised of cells pertaining to different lineages. SG stem/progenitor cells have been mostly shown to be residing in the intercalated ducts^37^. To understand SG development and devise treatments strategies for SG regeneration, it is important to identify stem/progenitor cells located in all the cellular compartments of SGs as there is no evidence of the generation of an entire functional gland from a single type of SG progenitor.

In the context of MSCs, existing studies have mostly focused on the characterization of cultured SG-MSCs derived from whole cell isolates, whose properties might be affected by *in vitro* expansion, and further might contain mixtures of cells of various lineages. Available reports on SG-MSCs have mostly focused on PAGs and SMGs with little information on SLGs being reported^17, 20, 38, 39^. Owing to the *in situ* and *in vitro* phenotypic and functional heterogeneity of MSCs derived from various tissues, identification of cell surface markers specifically recognizing MSCs in SGs is highly desirable. To our knowledge, our study is the first to present extensive *in situ*, *in vitro* and *in vivo* phenotypic and functional characterization of MSCs in human PAGs, SLGs and SMGs. We report that, (i) fetal SGs are rich in MSCs when compared to other tissues such as BM, (ii) CD34 is widely expressed by the stromal connective tissue progenitors of SGs, (iii) CD34^+^ cells express genes important for epithelial cell branching, clefting, cytodifferentiation and overall gland development, and (iv) CD34^+^ cell-derived MSCs possess potential to expand and differentiate into multiple mesodermal lineages *in vitro*, and when transplanted intraglandularly, can engraft in the stromal regions of immunodeficient mice.

Historically, CD34 has been regarded as a hematopoietic and endothelial cell marker^40^. Expression of CD34 on MSCs has been controversial, with the lack of CD34 expression being touted as a defining feature of MSCs^41^. Recently, multiple studies have demonstrated that depending on the tissue sources, most notably adipose and BM tissues, CD34 can also be expressed on freshly isolated MSCs^42, 43^. In our study, phenotypic expression analysis using a panel of stem/progenitor markers revealed that CD34 was highly expressed in human fetal PAGs, SLGs and SMGs. Furthermore, phenotypic and tissue localization assays ascertained that CD34^+^ cells expressed classical MSC markers, and were distributed in the stromal and connective tissues surrounding the acini and ducts of fetal SGs and adult SMGs. Our data suggested that, although a small population of CD34^+^ cells were found to co-express α-SMA and calponin, which might represent smooth muscle cells^44^, and were present in intralobular ducts^45^, the vast majority of CD34 expressing cells in SGs represent MSCs.

Our comparison of CD34^+^ populations between age-matched fetal SGs and BM revealed that the CFU-F colony forming potential of CD34^+^ cells was very less in BM as compared to fetal SGs. Additionally, >97% of BM CD34^+^ cells were of hematoendothelial origin. Our finding is in accordance with a study reporting that 5 × 10^−5^% to 0.1% of nucleated cells constitute the MSC pool in BM^46^. The differences in the properties of CD34^+^ cells between SGs and BM hint at the heterogeneity of MSCs across organs, and also suggest that not a single marker or phenotypic profile is truly representative of MSCs in all organs. Similar to the reports on adipose tissue MSCs, our results show that MSCs are more abundant in SGs than in BM; this highlights the differences in the abundance of MSCs depending on the tissue of origin^47^.

Recently, Banh *et al*., described CD34 as a stem cell marker in human adult SMGs and it was shown that CD34^+^ cells co-expressed CD44, c-kit and nestin^19^. We found that CD34^+^ cells co-expressed CD90, CD44 and nestin in fetal SGs, but lacked c-kit expression. Expression of c-kit and its function has been elegantly illustrated in murine SGs; however, existing studies are inconsistent regarding its expression in human SGs^18, 20, 48^. Our results suggest that in human SGs, CD34 and c-kit might represent different cell lineages.

Our study also focused on understanding the role of CD34^+^ cells as putative MSCs in the development, regeneration and maintenance of glandular homeostasis through performing comparative transcriptomic profiling of CD34^+^ and CD34^−^ cells isolated from human fetal SMGs (22 weeks’ gestation). MSCs have been shown to perform crucial roles in the development of embryonic murine SMGs at various stages of branching morphogenesis such as epithelial bud/ductal elongation, clefting and cell proliferation to form secretory acinar lobules, and cytodifferentiation^27, 49^. They perform these functions through providing molecular signals to the proliferating immature epithelial cells in the forms of ECM, basement membrane proteins, matrix metalloproteinases/proteases and growth factors. Functional roles of collagens and fibronectin have been shown to be very crucial in epithelial clefting and branching morphogenesis of SGs^14, 49–51^. Further, ECM assembly, degradation and remodeling are crucial events for the regulation of ECM turn over, cell-ECM interactions, release of growth factors, and for the migration of cells (e.g. neural crest cell migration)^52, 53^. Interestingly, we found that among the upregulated genes in the CD34^+^ population, majority of the genes belonged to, a) ECM/basement membrane proteins or encoding proteins containing domains homologous to ECM proteins, b) ECM assembly and remodeling, c) matrix metalloproteinases (MMPs) and ADAM family of proteases. The role of FGF signaling and its upstream regulator PDGF signaling pathways in early SG development and branching morphogenesis has been well established in murine studies^29, 30^. We found a significantly enriched expression of *FGF10* and *PDGFRA* in CD34^+^ cells. FGF10 signaling has been shown to facilitate the response of cells to EGFR signaling during primitive SG development^31^. Role of EGFR tyrosine kinases and their role in downstream ERK signaling has been previously shown to be important in branching morphogenesis through induction of cleft formation^54^. We found several genes belonging to the receptor tyrosine kinase family of proteins containing EGF/EGFR like motifs to be upregulated in CD34^+^ cells. Genes involved in the development of craniofacial structures, and organs with secretory and absorptive function similar to SGs such as lungs, kidneys, small intestine and mammary glands were found to be significantly enriched among CD34^+^ cells.

Among the downregulated genes in CD34^+^ cell population, most of the genes were found to be involved in hematopoietic, epithelial and neuronal pathways. Overall the gene expression analyses suggested that CD34^+^ cells represent MSCs in SGs and likely play a key role in organ development and regeneration through regulating critical functions including growth signals, cell-cell adhesion, cell migration, proliferation, branching and tissue remodeling.

Under culture conditions, CD34^+^ cells sorted from fetal SGs and BM proliferated rapidly, expressed MSC specific phenotypic markers and could be differentiated into multiple lineages. Little is known about the *in vivo* engraftment and reparative functions of human SG-MSCs as most studies have utilized BM or adipose tissue derived MSCs^55, 56^. However, owing to the heterogeneity of MSCs obtained from various sources, it is reasonable to hypothesize that tissue specific SG-MSCs could offer superior intrinsic functional abilities over MSCs from other sources in engrafting and repopulating SGs. Recently, two groups have demonstrated *in vivo* functionality of human SG-MSCs using murine models. One group showed that human adult PAG- and SMG-derived MSCs could repopulate and repair the function of SGs in rodents that were damaged with radiation. A second group illustrated that minor SG derived MSCs could engraft and function *in vivo* when transplanted intravenously into immunodeficient SCID mice^23, 39^. In this study we investigated and compared *in vivo* functions of human fetal PAG-, SLG- and SMG-MSCs with that of BM-MSCs.

Transplantation of cultured human SG-MSCs into mouse SMGs revealed that injected human cells could be detected at least until 30 days post cell injection, not only at the injected site but also in uninjected contralateral and ipsilateral SMGs and SLGs. Engraftment levels were poor when BM-MSCs were transplanted, suggesting *in vivo* functional differences between BM- and SG-MSCs. In mouse models of SG damage, the transplanted MSCs have been suggested to perform their migratory, homing and reparative functions under the influence of chemotactic factors released by injured tissues. Recently it was shown that under normal physiological conditions without SG damage, donor mouse BM stromal cells trafficked even to the uninjected glands when delivered intravenously, whereas cells remained primarily at the site of injection when transplanted intraglandularly^56^. Our results show that even in the absence of a significant damage-induced trigger, intraglandularly injected SG-MSCs were able to locate and redistribute into both injected and uninjected SMG and SLGs.

Analysis of the engrafted human cells showed that cultured fetal SG-MSCs that lost CD34 expression *in vitro* re-expressed CD34 *in vivo*, and contributed to the glandular stromal milieu. These findings indicate that standard MSC culture conditions fail to maintain the normal CD34^+^ phenotype on MSCs, but this phenomenon is subjected to reversal when cells are exposed to the *in vivo* microenvironment. This further confirms that glandular MSCs express CD34 under normal physiological conditions.

The clinical utility of MSCs has been impacted by the variability observed in their functions in various disease models and clinical trials. Targeted functional therapies employing MSCs from various tissue sources such as BM, Wharton’s jelly, umbilical cord blood, adipose cells, etc. suffer from tissue-dependent and donor-specific phenotypic and functional variations. Moreover, even though BM and adipose tissues are the most commonly used sources of MSCs, their isolation involves invasive, cumbersome extraction procedures. Our data show that MSCs are abundantly present in SGs as compared to BM, and in contrast to BM, SG-MSCs could engraft and be detected in mice SGs. These findings indicate that tissue specific SG-MSCs may have a better ability to regenerate damaged SGs in an organ-specific fashion over BM or adipose-derived MSCs. Moreover, our data reveal that CD34^+^ SG cells exhibit elevated expression of genes that are known to play key roles in the maintenance of homeostasis, and regulation of organ regeneration and repair. Taken together, our findings suggest that CD34^+^ SG cells could be a promising cellular source for treating a range of SG disorders and injuries. For instance, MSCs isolated and expanded from small portions of SGs of head and neck cancer patients prior to undergoing radiation therapy (RT) could be transplanted following RT to regenerate radiation-damaged SGs, leading to improved quality of life in xerostomia afflicted patients. The translational value of CD34^+^ SG cells within the context of regenerative and reparative therapies warrants further exploration.

In conclusion, we report that human fetal SGs harbor a rich and homogenous source of MSCs that can be isolated based on CD34 expression. The findings highlight the therapeutic potential of human fetal CD34^+^ cell-derived SG-MSCs in regenerative medicine, aiming at the clinical management of SG dysfunctions and disorders, as well as in bioengineering of artificial SGs.

## Materials and Methods

### Collection of tissues and isolation of cells

This study was approved by the University of California at San Francisco Institutional Review Board (UCSF IRB), and all methods were performed in accordance with the relevant guidelines and regulations. Paired PAG, SLG, SMG and matched BM tissues (n = 10) were obtained from 16 to 24 weeks’ gestation fetuses. Fetal specimens were obtained from San Francisco General Hospital, CA, USA. After the decision to have an abortion was made, women were informed of the possibility to donate the specimens for research. Women had the choice to decline or provide written consent to donate the tissue. Written informed consent was obtained by clinic staff and not by the researchers involved in this study in order to ensure anonymity of the donors. Specimens were collected without identifying information and no identifying information is contained within this report. Specimens, collected shortly after termination of the pregnancy, were dissected under a Leica MZ16 F stereomicroscope (Leica Microsystems, Buffalo Grove, IL).

Adult SMGs (n = 3; 31–64 yrs), from healthy non-irradiated donors undergoing neck resection were collected from the Department of Otolaryngology and Head-Neck surgery division of the University of San Francisco (UCSF), CA, USA. According to UCSF IRB’s policies and federal guidelines for the research involving unidentifiable biological specimens, UCSF IRB review is not required. The tissues were not collected specifically for the purposes of this study, but were residual clinical specimens which were made available for research use. No identifying information is contained within this report regarding these specimens.

Cells were isolated by digestion of SG tissues with collagenase IV (1 mg/ml) (Life Technologies, Grand Island, NY) and dispase II (1 U/ml) (Roche, South San Francisco, CA) at 37 °C for 1 to 2 hours. Single cell suspensions were prepared by filtering the digested tissue lysate through a 40 µM filter (Millipore, Billerica, Massachusetts). BM cells were isolated by flushing of the marrow canal from long bones^57^. Following density gradient centrifugation, BM mononuclear cells (MNCs) were collected from the light-density (≤1.077 g/ml) fraction.

### Cell sorting

Fetal PAG, SLG and SMG single cells were stained with CD34 APC/FITC antibodies, followed by anti-APC/FITC microbeads. Fetal BM-MNCs were stained with CD271 FITC and anti-FITC microbeads. Further stained cells were passed through a LS + separation column attached to a Midi-MACS separation unit (Miltenyi Biotech, Auburn, CA) to obtain purified magnet-retained positively stained and magnet-depleted negatively stained cells. The cells were run on a BD LSR II flow cytometer (BD Biosciences, San Jose, CA) and found to be 88% to >90% pure and >95% viable. Propidium iodide was used to differentiate between live and dead cells. The data was further analyzed using FlowJo software (FlowJo, Ashland, OR).

### Flow cytometric analysis

Freshly isolated fetal SG cells and BM-MNCs and *in vitro* cultured CD34^+^ cell derived SG and BM-MSCs were stained with a panel of monoclonal antibodies (mAbs) specific for various lineage specific stem/progenitors (see Table S1) and, were analyzed by flow cytometry as described above.

### Culture of MSCs

MSCs were derived from purified CD34^+^ cells of fetal PAGs, SLGs, SMGs and BM-MNCs by plating on tissue-culture treated plastic 10 cm dishes (Cellstar, St. Louis, MO) with MSC growth medium containing α-MEM (Life Technologies, Grand Island, NY) supplemented with 10% fetal bovine serum (Life Technologies, Grand Island, NY) and gentamicin (50 μg/ml) (Sigma Aldrich, St. Louis, MO) and were cultured in a 37 °C humidified CO_2_ incubator until confluent. At 70–80% confluence the adherent cells were passaged by dissociating with 0.1% trypsin (Life Technologies, Grand Island, NY).

### CFU-F assay

Purified CD34^+^ and CD34^−^ cell fractions were subjected to colony-forming unit fibroblast (CFU-F) assay by plating 1000 cells/well in 6-well plates (Cellstar, St. Louis, MO) containing MSC growth medium. Cells were cultured in a 37 °C fully humidified 5% CO_2_ incubator for 14 days. Colonies (≥50 fibroblastoid cells) were stained with Giemsa stain. All the experiments were done in duplicates and repeated three times.

### Multilineage differentiation of MSCs

5 × 10^5^ fetal SG and BM-MSCs (passages 3 to 12) were cultured in StemPro osteogenic, adipogenic and chondrogenic differentiation inducing media (Life Technologies, Grand Island, NY). For osteogenic and adipogenic differentiation cells were grown for 7–14 days, and differentiation was confirmed by von Kossa and Oil Red O stainings, respectively. After 21 days of culture, chondrocyte differentiation was analyzed by Alcian blue staining. All the experiments were done in duplicates and repeated three times.

### Immunofluorescence and immunohistochemistry

For immunofluorescence, cultured MSCs were fixed in 4% paraformaldehyde (PFA) and processed as described previously^58^. Cells were stained with primary and secondary antibodies listed in Table S1. For immunohistolocalization assays, freshly collected fetal SGs and adult SMGs were fixed and embedded as described previously^59^. 10 µm cryostat sections were subsequently stained with primary and secondary antibodies (Table S1) and were counterstained with ProLong® Gold antifade reagent with 6-diamidino-2-phenylindole (DAPI) (Life Technologies, Grand Island, NY). Images were captured on Leica CTR6500 (Leica Microsystems, Buffalo Grove, IL) and EVOS epifluorescence microscopes (Thermo Fisher Scientific, Waltham, MA). Images were further analyzed using NIH-ImageJ software (NIH, Bethedsa, Maryland). Quantification of the images was done using NIH-ImageJ software to analyze 10 to 15 random images at 20x magnification from ≥3 fetal and adult SMG sections.

### RNA sequencing of CD34 cell transcriptome of fetal SMGs

Total RNA was extracted from sorted CD34^+^ and CD34^−^ cells obtained from freshly collected 22 weeks’ gestation fetal SMGs, using RNeasy mini kit (Qiagen, Venlo, Limberg). RNA samples were quantified using Qubit 2.0 Fluorometer (Life Technologies, Carlsbad, CA, USA) and RNA integrity was checked with 2100 Bioanalyzer (Agilent Technologies, Palo Alto, CA, USA). RNA sequencing library preparation used the NEBNext Ultra RNA Library Prep Kit for Illumina by following manufacturer’s recommendations (NEB, Ipswich, MA, USA). Briefly, mRNA was first enriched with Oligod(T) beads. Enriched mRNAs were fragmented for 15 minutes at 94 °C. First strand and second strand cDNA were subsequently synthesized. cDNA fragments were end repaired and adenylated at 3’ends, and universal adapter was ligated to cDNA fragments, followed by index addition and library enrichment with limited cycle PCR. Sequencing libraries were validated using a DNA Chip on the Agilent 2100 Bioanalyzer (Agilent Technologies, Palo Alto, CA, USA), and quantified by using Qubit 2.0 Fluorometer (Invitrogen, Carlsbad, CA) as well as by quantitative PCR (Applied Biosystems, Carlsbad, CA, USA).

The sequencing libraries were multiplexed and clustered onto a flowcell. After clustering, the flowcell was loaded on the Illumina HiSeq 2500 instrument according to manufacturer’s instructions. The samples were sequenced using a 1 × 50 bp Single Read (SR) configuration. Image analysis and base calling were conducted by the HiSeq Control Software (HCS) on the HiSeq 2500 instrument. Raw sequence data (.bcl files) generated from Illumina HiSeq 2500 was converted into fastq files and de-multiplexed using Illumina bcl2fastq v 1.8.4 program. One mis-match was allowed for index sequence identification. The sequencing reactions were conducted at GENEWIZ, LLC. (South Plainfield, NJ, USA).

### RNA isolation and quantitative gene expression analysis

Total RNA was extracted from multilineage differentiated SG-MSCs, freshly isolated CD34^+^ and CD34^−^ SMG cells, CD271^+^ BM cells using RNeasy mini kit (Qiagen, Venlo, Limberg). cDNA was synthesized using a reverse transcription kit (Applied Biosystems, Grand Island, NY). Age of all the fetal tissues used ranged from 16–22 weeks’ gestation. Quantitative real-time PCR (Q-PCR) was performed using gene specific primers using Fast SYBR Green master mix (Applied Biosystems, Grand Island, NY) in a Viia7 real-time PCR system (Life Technologies, Grand Island, NY). Sequences of the primers have been provided in Table S2. MSCs ranging from passages 3 to 12 were used for this analysis. All samples were run in triplicates and the expression levels were calculated by minimal cycle threshold (Ct) values normalized to the reference expression of ubiquitin C (*UBC*) gene in each sample.

### Xenotransplantation and analysis of engraftment

Human cells were transplanted into the right SMGs of immunodeficient NSG mice (≥8 weeks of age), which were bred at our institute with founders purchased from Jackson Laboratory (Bar Harbor, ME). MSCs derived from enriched CD34^+^ cells of PAGs, SLGs, SMGs and BM-MNCs were transplanted intraglandularly into the right SMG of NSG mice. Cells were injected at three doses viz. 0.5, 1 and 2 million cells/mouse, and in each group a total of 10 mice were included. Mice were sacrificed after 15 and 30 days (n = 5 in each group) of cell transplantations, and the paired SMG and SLG tissues were analyzed for the engraftment of transplanted human cells. A portion of each pair of the harvested SMGs and SLGs was processed for immunohistochemistry as described previously^59^. The remaining parts of the harvested tissues were digested with collagenase IV and cells were analyzed by flow cytometry. Antibodies used are listed in (Table S1).

Genomic DNA was extracted from mouse SLGs, SMGs, liver and lungs using DNeasy Tissue Kit (Qiagen, Venlo, Limberg). Quantitative real-time PCR was done using human specific, *HLA-39B* and mouse specific, *GAPDH* primers (Table S2) using Fast SYBR Green master mix (Applied Biosystems, Grand Island, NY) in a Viia7 real-time PCR system (Life Technologies, Grand Island, NY). We have selected *HLA-A-39B* for this study after confirming its specificity to detect human cells in murine background, after testing for other human specific genes such as, *HLA-A-54* and *CMT1A* ^36^. Mouse glyceraldehyde phosphate dehydrogenase (*GAPDH*) primers were used to verify the integrity of mouse DNA. ~15,000 cells equivalent genomic DNA (100 ng) was used for the assay. Human DNA was mixed with mouse DNA to generate standard curve starting with 15 cells equivalent human DNA (in a background of 15,000 cells equivalent mouse DNA) to 15,000 cells equivalent human DNA (in a background of 15,000 cells equivalent mouse DNA). Subsequently number of engrafted human cells in mice SLGs and SMGs could be extrapolated using the standard curves. To decrease intratest variability at very low concentrations of DNA, three samples were used for each dilution in standard curves and samples were run in triplicates all test conditions.

### Statistics

Data are presented as mean ± SEM. The nonparametric Mann-Whitney t-test was used for unpaired comparisons. Means of multiple groups were compared by one-way analysis of variance (ANOVA) using Tukey’s multiple comparison test. P < 0.05 was considered significant. Analyses were performed using GraphPad Prism (GraphPad Software, Inc. La Jolla, CA). For RNAseq analyses, the preprocessed reads were mapped using Tophat to the reference genome hg19^60, 61^. Then gene level expression quantification in FPKM (Fragments Per Kilobase of transcript per Million mapped reads) were calculated using Cufflinks suite including Cufflinks, Cuffmerge, Cuffquant and Cuffnorm^62^. False discovery rates (FDR) were computed using the Benjamini-Hochberg procedure to adjust for multiple comparisons in the RNA-seq data^63^. The heatmaps were generated using standardized Z-scores (created using the R statistical package).

## Electronic supplementary material

Supplementary files
