# Supplementary material for: Identification and characterization of a rich population of CD34+ mesenchymal stem/stromal cells in human parotid, sublingual and submandibular glands
Source: Sci Rep. 2017 Jun 14;7:3484. doi: 10.1038/s41598-017-03681-1 (PMC5471181; doi:10.1038/s41598-017-03681-1)
Supplement: Supplementary file 1 — Supplementary files [file 41598_2017_3681_MOESM1_ESM.pdf]

Identification and characterization of a rich population of CD34$^+$ mesenchymal stem/stromal cells in human parotid, sublingual and submandibular glands

Padma Priya Togarrati[1], Robson T Sasaki[2], Mohamed Abdel-Mohsen[1,3,4], Nuntana Dinglasan[1], Xutao Deng[1], Shivani Desai[1], Elaine Emmerson[5], Elizabeth Yee[1], William R Ryan[6], Marcelo C P da Silva[2], Sarah M Knox[5], Satish K Pillai[1,7], Marcus O Muench[1,7]*

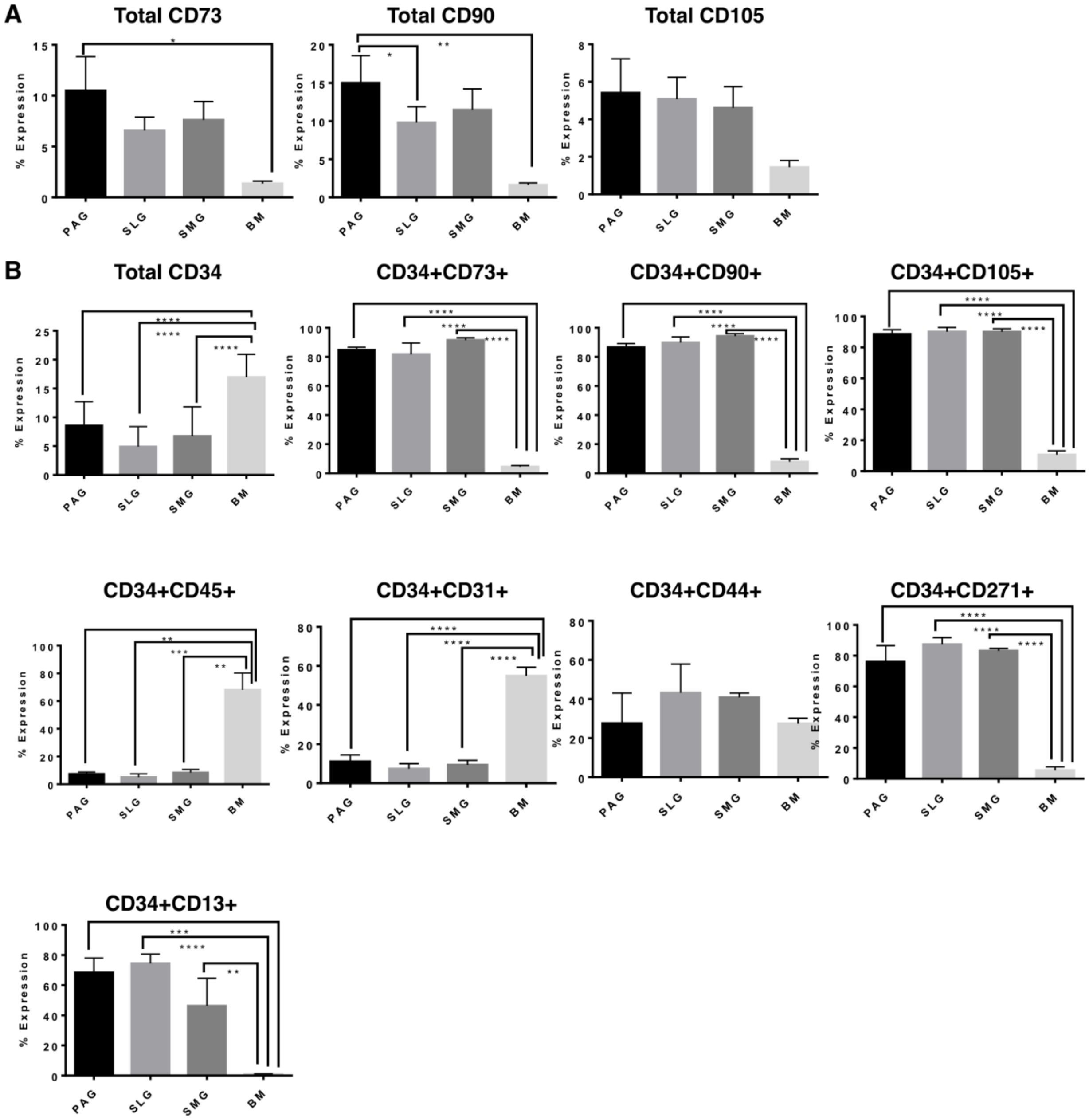

Figure S1: Flow cytometric analysis of stem/progenitor cell surface marker expressions in fetal PAGs, SLGs, SMGs and BMs (N=3 to10) A) Expressions of total CD73, CD90, CD105 B) Expression of total CD34; CD73, CD90, CD105, CD45, CD31, CD44, CD271, and CD13 on total CD34+ cells. Error bars represent mean±SEM.

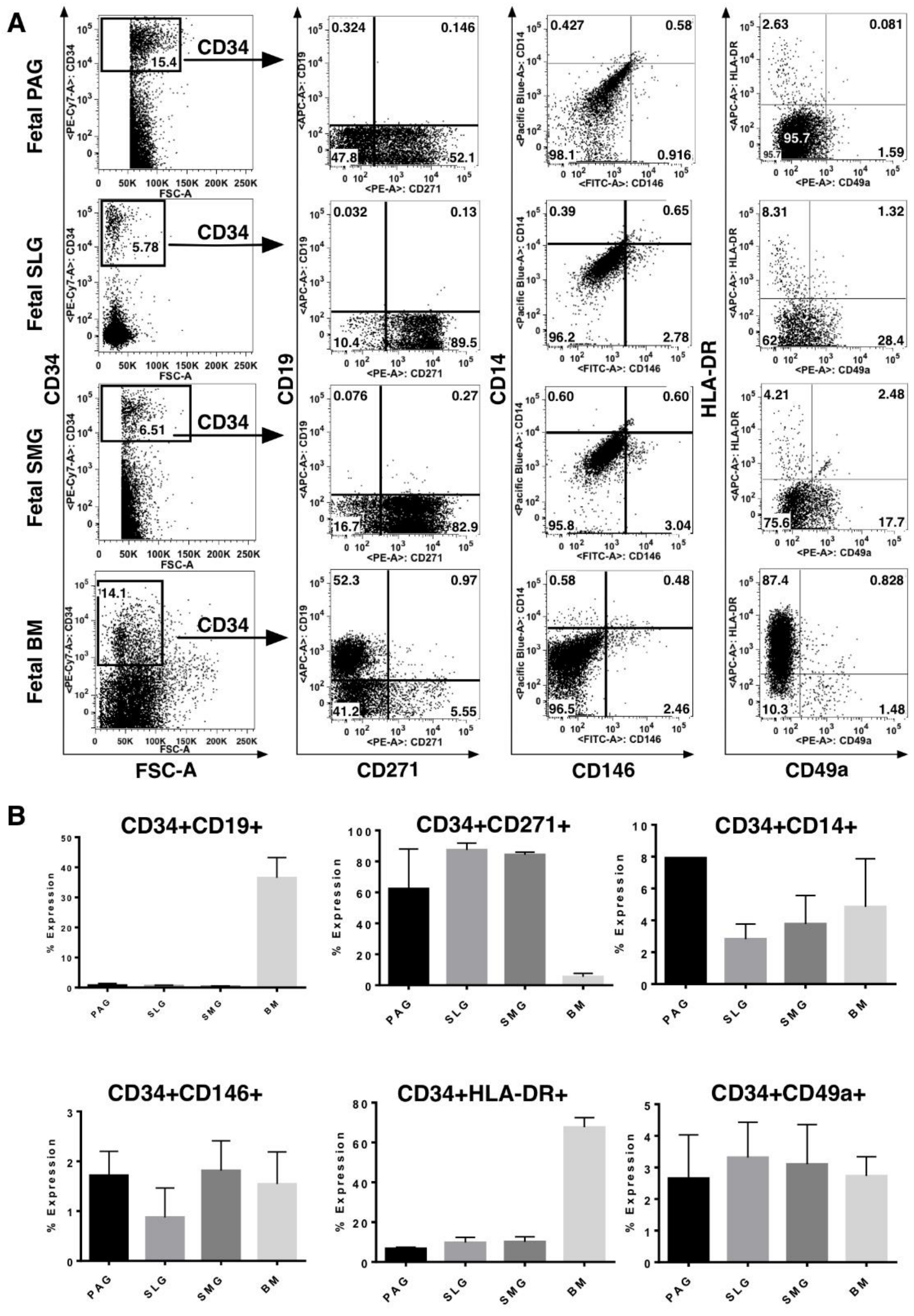

Figure S2: A) and B) Representation of expression of CD19, CD271, CD14, CD146, HLA-DR and CD49a on total CD34+ cells in fetal PAGs, SLGs, SMGs and BMs (N=3 to 10). Error bars represent mean±SEM.

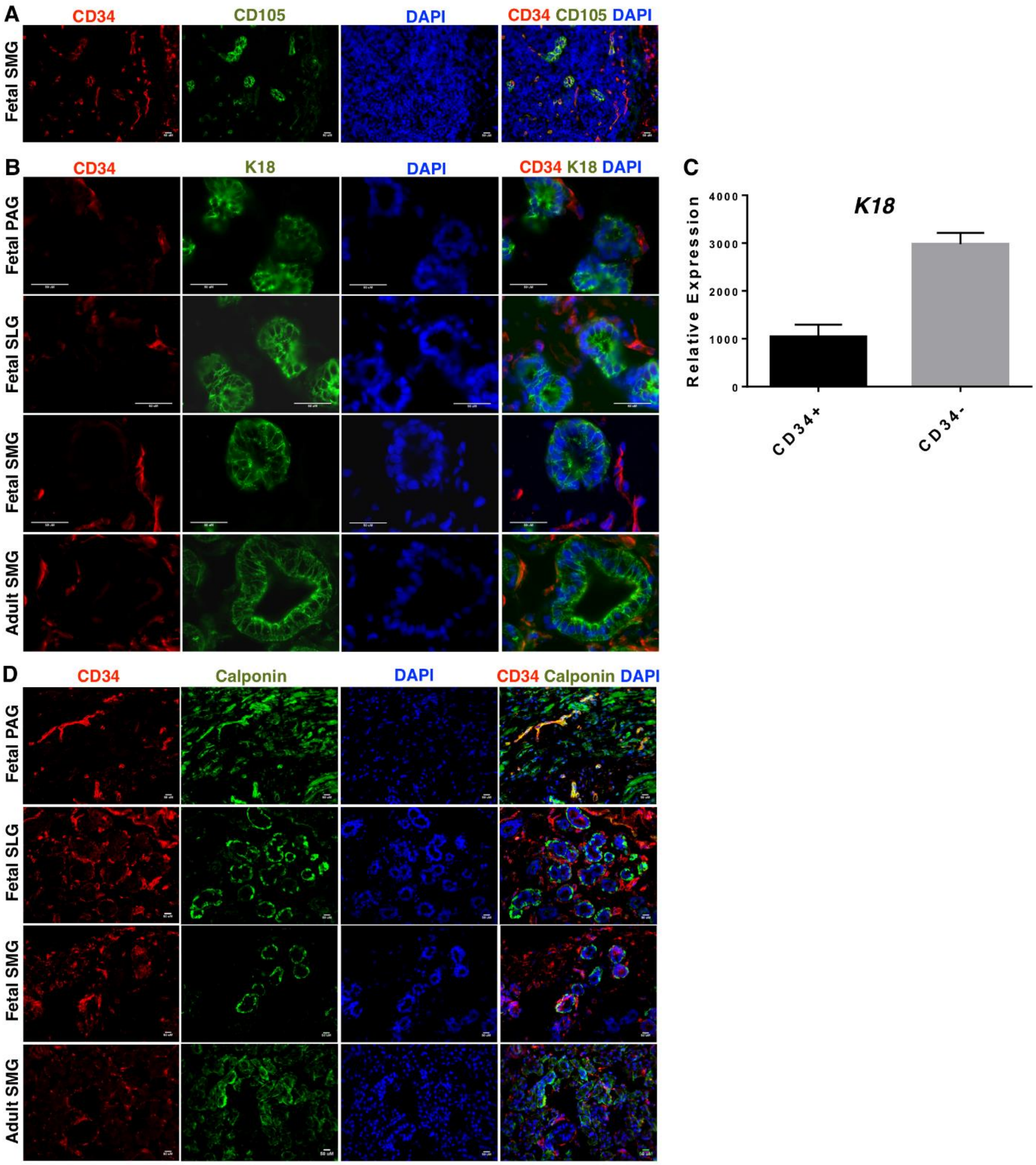

Figure S3: A) Expressions of CD34 (red) and CD105 (green) in fetal SMG **B)** Expressions of CD34 (red) and K18 (green) in fetal PAG, SLG, SMG, and adult SMG. C) Analysis of K18 expression through quantitative real-time PCR. **D)** Expressions of CD34 (red) and calponin (green) in fetal PAG, SLG, SMG, and adult SMG. Nuclei was stained with DAPI (Blue). Scale bars represent 50μM**.**

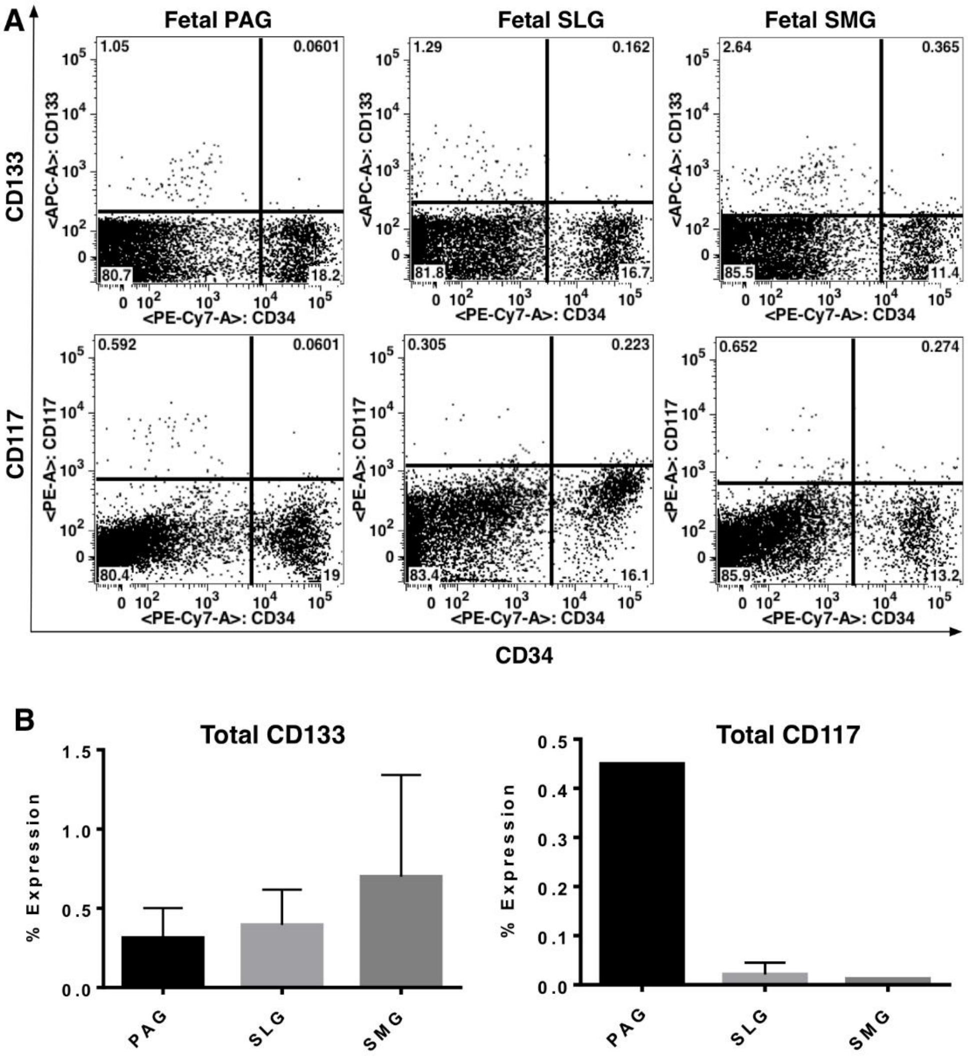

Figure S4: A) and **B)** Expressions of CD133 and CD117 in fetal PAGs, SLGs and SMGs (N=3 to 5). Error bars represent mean±SEM.

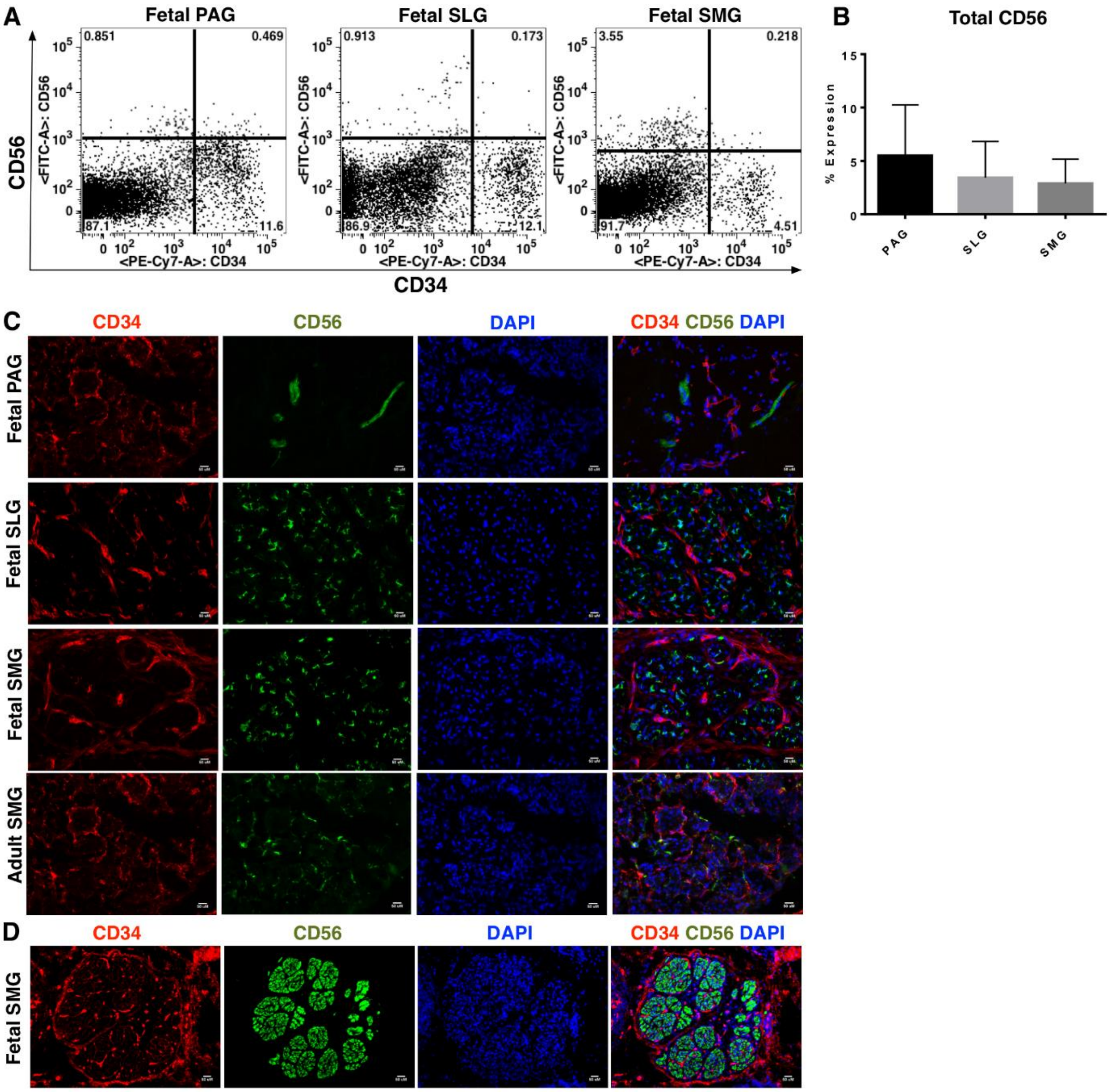

Figure S5: A) and B) Demonstration of expressions of CD56 and CD34 in fetal PAGs, SLGs and SMGs. Error bars represent mean±SEM. **C)** Immunohistolocalization of CD34 (red) and CD56 (green) in fetal SGs and adult SMG. **D)** Immunohistolocalization of CD56 (green) in a nerve bundle of fetal SMG. Nuclei was stained with DAPI (Blue). Scale bars represent 50μM

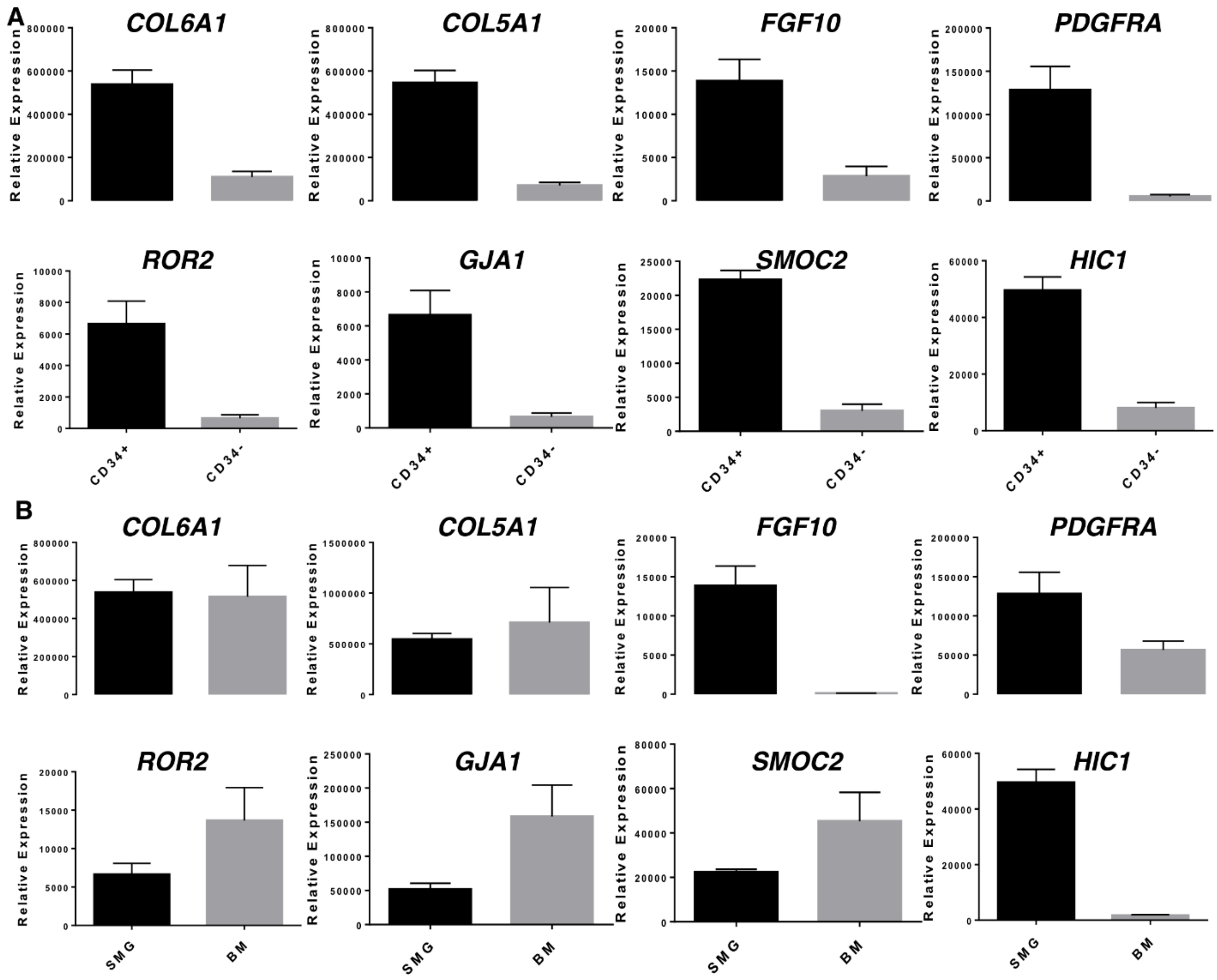

Figure S6: A) Validation of high expression of genes in CD34+ SMG cells through quantitative real-time PCR. **B)** Comparative gene expression analysis between CD271+BM and CD34+ SMG cells

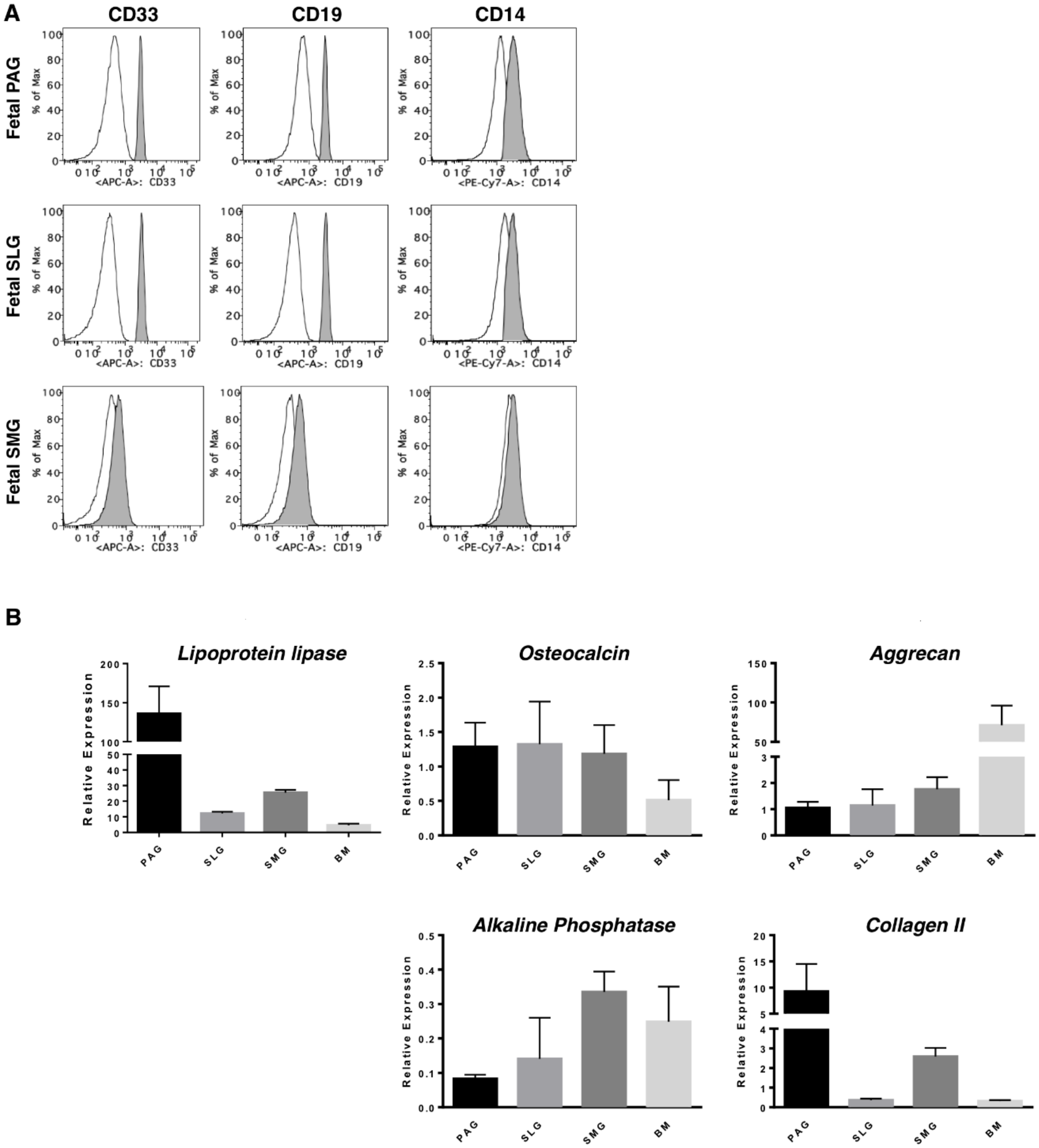

Figure S7: A) Representation of expressions of CD33, CD19 and CD14 on CD34+ cells derived cultured SG-MSCs. Grey bars represent isotype controls. **B)** Quantitative gene expression analyses of multilineage differentiations of CD34+ cells derived PAG-MSCs (15, 18, 23 weeks' gestation), SLG-MSCs (16, 19 weeks' gestation), SMG-MSCs (18, 19 weeks' gestation), and total BM-MNC derived MSCs (21, 22 weeks' gestation) into adipocyte (*lipoprotein lipase* gene), osteocyte (*osteocalcin* and *alkaline phosphatase* genes) and chondrocyte (*aggrecan* and *collagen type II* genes) cells. Passages of all MSCs were between 3 to 12. Error bars represent mean±SEM

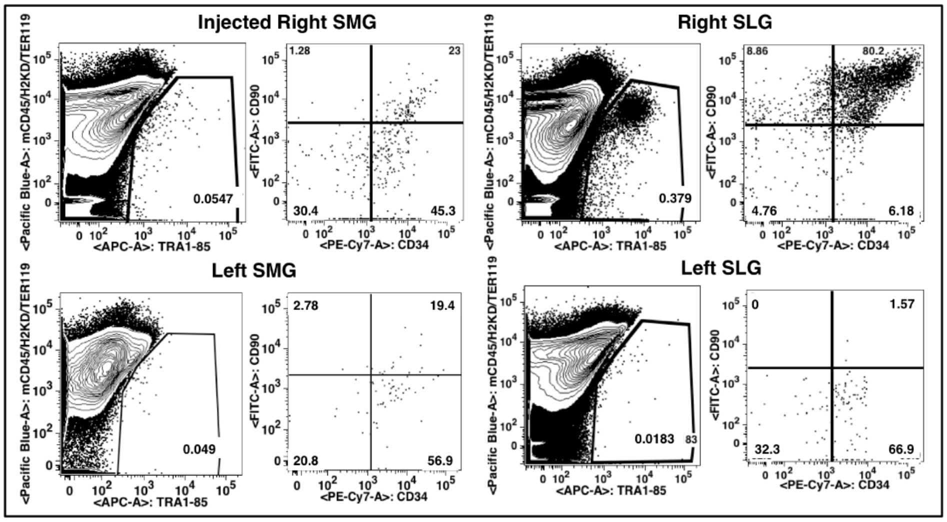

Figure S8: Representative pattern of engraftment of human cells (after 30 days of cell transplantation) in a NSG mouse when transplanted with CD34+ cell derived SG-MSCs into the right SMG.

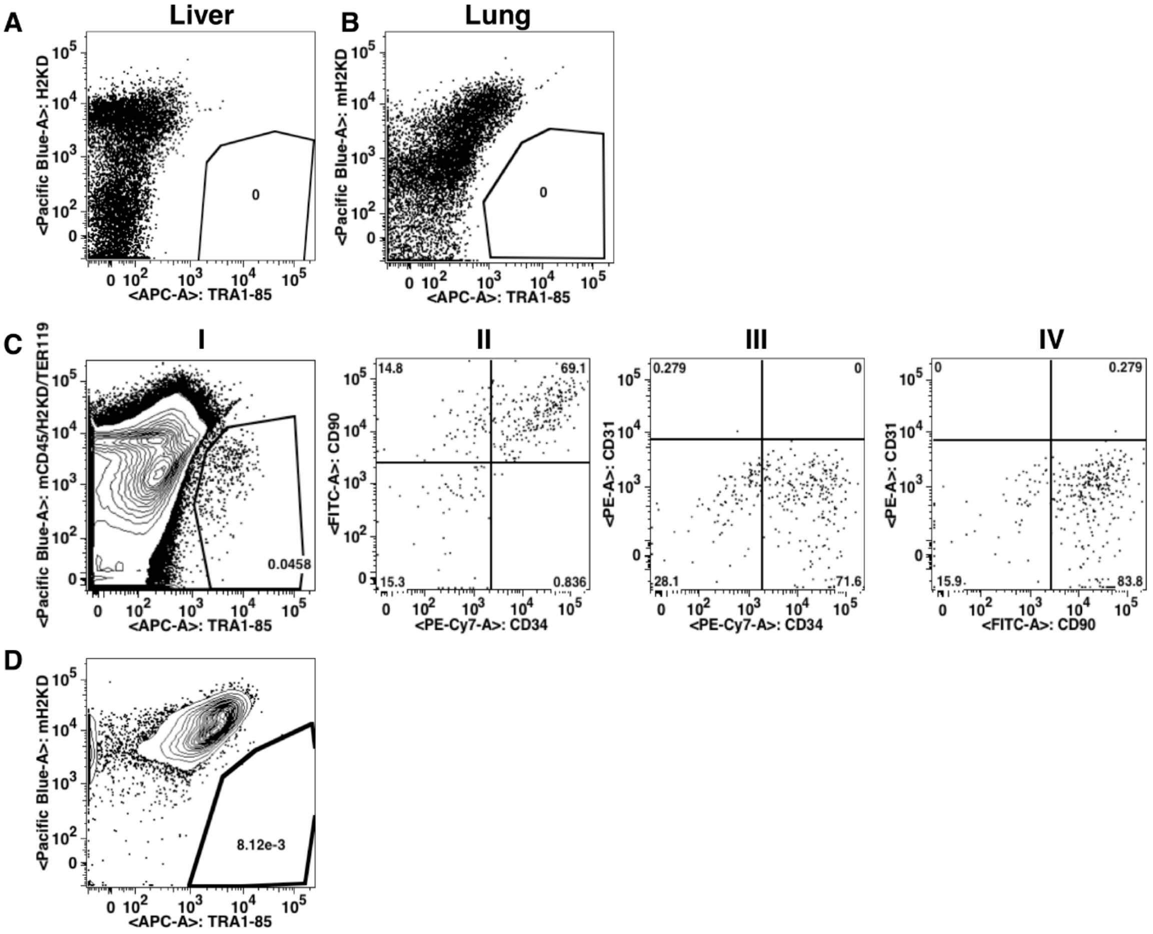

Figure S9: Engraftment analysis of transplanted CD34+ cell derived SG-MSCs and total BM-MSCs in NSG mice. **A and B)** Analysis of engraftment of human cells in mouse liver and lung, respectively. **C)** Analysis of CD31 on engrafted human cells. **D)** Analysis of engraftment of BM-MSCs.

Table S1. **Antibody reagents and their sources used in the study.**

Antibody	Fluorophore	Company	Catalog No.	Clone	Isotype
**Antibodies recognizing human antigens**					
CD13	APC	BioLegend	301705	WM15	IgG1
CD14	PB	Biolegend	325615	HCD14	IgG1
CD19	APC	BioLegend	302211	HIB19	IgG1
CD29	PE	BD Pharmingen	555443	MAR4	IgG1
CD31	FITC	Biolegend	303109	WM59	IgG1
CD31	PE	Biolegend	303105	WM59	IgG1
CD33	APC	Biolegend	303407	WM53	IgG1
CD34	PE-Cy7	Biolegend	343515	VMA27	IgG1
CD34	FITC	BD Biosciences	555821	581	IgG1
CD44	PE	BioLegend	338807	BJ18	IgG1
CD45	PB	BioLegend	304021	HI30	IgG1
CD45	APC	Biolegend	304037	HI30	IgG1
CD49a	PE	BioLegend	328303	TS2/7	IgG1
CD56	FITC	Biolegend	318303	HCD56	IgG1
CD73	PE	BioLegend	344003	AD2	IgG1
CD90	FITC	BioLegend	328107	5E10	IgG1
CD90	PE	Biolegend	328109	5E10	IgG1
CD105	APC	BioLegend	323207	43A3	IgG1
CD105	PE	Life Technology	MHCD10504	SN6	IgG1
CD133	APC	Miltenyi Biotec	130-090-826	AC133	IgG1
CD146	FITC	Miltenyi Biotec	130-092-851	541-10B2	IgG1
CD147	APC	eBiosciences	17-1472-42	8D12	IgG1
CD271	PE	BD Biosciences	557196	C40-1457	IgG1
HLA-DR	APC	BioLegend	307609	L243	IgG2a
**Non-specific antibodies for isotype control**					
IgG1	APC	BioLegend	400120	MOPC-21	

Antibody	Fluorophore	Company	Catalog No.	Clone	Isotype
IgG1	FITC	BioLegend	400108	MOPC-21	
IgG1	PE	BioLegend	400114	MOPC-21	
IgG1	PE-Cy7	BioLegend	400126	MOPC-21	
IgG2a	APC	BioLegend	400220	MOPC-173	
IgG2a	FITC	BioLegend	400208	MOPC-173	
IgG2a	PE	BD Biosciences	559319	G155-178	
IgG2b	PE	BD Biosciences	555743	27-35	
**Antibodies recognizing mouse antigens**					
CD45	Pacific Blue	BioLegend	103126	30-F11	
H2-K^d	Pacific Blue	BioLegend	116616	SF1-1.1	IgG2a
H2-K^d	AF647	BioLegend	116612	SF1-1.1	IgG2a
TER-119	Pacific Blue	BioLegend	116232	TER-119	
**Secondary antibodies**					
IgG1 goat anti-mouse	AF488	Life Technology	A21121		
IgG1 goat anti-mouse	AF568	Life Technology	A21124		
IgG2a goat anti-mouse	AF568	Life Technology	A21134		
IgG2b goat anti-mouse	AF568	Life Technology	A21144		
donkey anti-sheep	AF488	Life Technology	A11015		
rabbit anti-goat	FITC	Sigma Immuno Chemicals	F-2016		
**Primary antibodies**					

Antibody	Fluorophore	Company	Catalog No.	Clone	Isotype
CD31	unconjugated	Cell signaling tech.	3528	89C2	IgG2b
CD34	unconjugated	BioLegend	343602	561	IgG2a
CD56	Purified	Becton Dickinson	347740	MY31	IgG1
CD105	unconjugated	Life Technology	MHCD10500	SN6	IgG1
CD133	unconjugated	Miltenyi Biotec	130-090-422	AC133	IgG1
SMA	Cy3	Sigma	C6198	1A4	IgG2a
K5	unconjugated	abcam	ab17130	XM26	IgG1
Calponin	unconjugated	Millipore	ABT129	Polyclonal	
Vimentin	unconjugated	Cell signaling tech.	5741	D21H3	IgG
Human mitochondrial Ab	unconjugated	Millipore	MAB1273	113-1	IgG1
Human nuclei Ab	unconjugated	Millipore	MAB1281	235-1	IgG1
EpCAM-1 (CD326)	conjugated	Miltenyi Biotec	130-080-301	caa7-9G8	IgG1
K18	unconjugated	Invitrogen	558077A	5D3	IgG1
Nestin	unconjugated	Santa Cruz Biotechnology	SC-23927	10c2	IgG1

Table S2: List of sequences of gene-specific primers used in Q-PCR assay

Human GAPDH	5’ AGG GCT GCT TTT AAC TCT GGT 3’	5’ CCC CAC TTG ATT TTG GAG GGA 3’
Lipoprotein lipase	5’ AAA GCC CTG CTC GTG CTG AC 3’	5’ TAA ACC GGG CCA CAT CCT GT 3’
Osteocalcin	5’ TGC AGA GTC CAG CAA AGG TG 3’	5’ GAT GTG GTC AGC CAA CTC GTC 3’
Alkaline phosphatase	5’ CTG GAC CTC GTT GAC ACC TG 3’	5’ GAC ATT CTC TCG TTC ACC GC 3’
Aggrecan	5’ CCT CTG GAC AAC CAG GTG TT 3’	5’ AAA CCA GGT CAG GGA CTC CT 3’
Collagen II	5’ ACC CCA ATC CAG CAA ACG TT 3’	5’ ATC TGG ACG TTG GCA GTG TTG 3’
COL6A1	5’ TCA GAA TAG TGA TGT GTT CGA CGT T 3’	5’ AGC AAC ATG GAT ATG GTT CAG AAA 3’
COL5A1	5’ GGC TCC CGA GAG CAA CCT 3’	5’ CGG GAC ACT CAC GAA CGA A 3’
FGF10	5’ ATG TCC GCT GGA GAA AGC TA 3’	5’ CCC CTT CTT GTT CAT GGC TA 3’
PDGFRA	5’ CTG GGT TTC CAT CCT TGA G 3’	5’ TAG TAG GCT TCC TGC GTG G 3’
ROR2	5’GTG CGG TGG CTA AAG AAT GAT 3’	5’ ATT CGC AGT CGT GAA CCA TAT T 3’
GJA1	5’ TAC CAT GCG ACC AGT GGT GCG CT 3’	5’ GAA TTC TGG TTA TCA TCG GGG AA 3’
SMOC2	5’ AGG TCC TTC CGT CAG ATG C 3’	5’ AGA AGT TCT CGG CGC TCA C 3’
HIC1	5’ CGA CGA CTA CAA GAG CAG CAG C 3’	5’ CAG GTT GTC ACC GAA GCT CTC 3’
HLA-A-39B	5’ CCA AGA AAG AAA GAA CCA ATA GCA 3’	5’ GAG CCA ATT AGC CCA ATA AAT CAC 3’
Mouse GAPDH	5’ GGG TGG AGC CAA ACG GGT C 3’	5’ GGA GTT GCT GTT GAA GTC GCA 3’
Human UBC	5’ CGG TGA ACG CCG ATG ATT AT 3’	5’ ATC TGC ATT GTC AAG TGA CGA 3’
